# Interpretable Small-Sample Deep Learning with Approximate Attribution Enhancement for Atomically Precise Gold Nanoclusters Synthesis

**DOI:** 10.3390/molecules31162808

**Published:** 2026-08-12

**Authors:** Ziyue You, Yitong Qin, Yubing Gao, Zhengzhi Mu, Fu Liu, Shuzhi Sam Ge

**Affiliations:** 1 College of Communication Engineering, Jilin University, Changchun 130022, China; soloyao0603@163.com; 2 Department of Electrical and Computer Engineering, National University of Singapore, Singapore 117583, Singapore; samge@nus.edu.sg; 3 State Key Laboratory of Intelligent Manufacturing Equipment and Technology, Huazhong University of Science and Technology, Wuhan 430074, China; drqyt@hust.edu.cn; 4 School of Physics, Changchun University of Science and Technology, Changchun 130022, China; gaoyb@cust.edu.cn; 5 Key Laboratory of Bionic Engineering, Ministry of Education, Jilin University, Changchun 130022, China; 6 Institute of Structured and Architected Materials, Liaoning Academy of Materials, Shenyang 110167, China

**Keywords:** gold nanoclusters, small-sample learning, graph convolutional neural network, data augmentation, interpretable machine learning

## Abstract

Small experimental datasets make synthesis-condition modelling particularly sensitive to overfitting and data leakage. Here, a graph convolutional neural network (GCNN) was coupled with approximate attribution enhancement (AAE) and evaluated on 54 gold-nanocluster synthesis records using a strict fold-local pipeline. All preprocessing, supervised representation learning, augmentation, model selection, and calibration were confined to each training fold, and metrics were calculated only on untouched original records. The strongest no-augmentation model achieved a Matthews correlation coefficient (MCC) of 0.729. With 20,000 fold-local AAE samples, Logistic Regression reached an accuracy of 0.864, an F1 score of 0.862, and an MCC of 0.758. The corresponding MCC values decreased to 0.579, 0.526, and 0.652 under leave-one-ligand-out, leave-one-publication-out, and leave-region-out evaluation, respectively, indicating that extrapolation beyond represented chemistry remained more difficult than random-fold interpolation. Feature, label-weight, augmentation-baseline, covariance, calibration, and stability analyses consistently identified ligand aromaticity, temperature, and HAuCl_4_ concentration as the most influential factors. An interpretable analysis of a 34-record aqueous Au_25_ subset further produced condition rules and a pH–temperature map, while nine literature formulations provided an external audit within the represented domain. The results position AAE as a local regularisation strategy for data-limited synthesis modelling rather than as a source of new chemical information.

## 1. Introduction

Atomically precise gold nanoclusters (Au NCs) with exact nuclearity and well-defined structures have attracted broad interest in catalysis, bioimaging, optoelectronic sensing, and quantum materials, owing to their discrete electronic energy levels, tunable optical properties, and high thermal and chemical stability [1,2,3]. Their properties are exquisitely sensitive to nuclearity and ligand structure, so that even subtle compositional changes translate into pronounced differences in optical and catalytic behaviour [4]; prototypical clusters such as Au_25_(SR)_18_ form reproducibly only when the reducing agent, ligand, pH, and temperature are tuned in concert [5]. Consequently, obtaining Au NCs with a specific nuclearity under controllable conditions remains a central challenge in precision nanomaterial synthesis [6], and, from an engineering standpoint, it is naturally posed as a design-optimisation problem over a high-dimensional, weakly observed condition space.

The Brust-Schiffrin method is a common route to Au NCs and typically involves stepwise reduction from Au(III) and Au(I) to Au(0) and self-assembly of Au(I)-SR precursor species [7]. Although operationally simple, the reaction system couples reduction kinetics, thiol deprotonation, coordination polymerisation, and the competition between nucleation and growth, so that the formation of monodisperse Au NCs is highly sensitive to the solvent, ligands, the molar ratio of HAuCl_4_ to reducing agent (RA), pH, and temperature [8,9]; controlled syntheses confirm that the chemical nature of the ligand, the pH, and the temperature jointly dictate cluster size and emission [10]. For highly stable Au_25_ clusters, the feasible synthesis window is strongly nonlinear and extremely narrow, making empirical tuning heavily dependent on extensive trial-and-error [11]. Moreover, most reported studies scan only a few variables within similar reaction pathways, producing sparse observations in a high-dimensional condition space and limiting any systematic understanding of how multiple parameters jointly determine the product. A predictive, interpretable computational model is therefore highly desirable as a decision-support tool for experiment design.

In recent years, computer-aided synthesis planning (CASP) and machine learning (ML) have developed rapidly and demonstrated strong potential for reaction prediction, condition optimisation, and pathway search in complex chemical systems [12,13,14,15]. Large-scale frameworks have been established for organic synthesis, drug discovery, and reaction-condition prediction, including template-free retrosynthesis networks and transformer-based reaction models [16,17,18]. However, these models generally rely on thousands to millions of high-quality labelled samples, and even data-efficient transformers presuppose pre-training corpora that do not exist for nanocluster chemistry [19]. Such paradigms cannot be transferred directly to nanocluster synthesis, where experimental samples are extremely limited and variable interactions are complex [20,21,22]; comprehensive QSAR analyses further warn that deep models readily overfit and forfeit interpretability on small datasets unless explicit applicability-domain and regularisation safeguards are imposed [23]. Even within the nanomaterials field, existing ML studies have focused mainly on structure prediction, formation-energy calculation, or fitting of electronic properties, such as stability prediction for Au clusters and the construction of neural-network potentials [24,25,26], with growing use of (quasi-)SMILES representations to encode nanomaterial building blocks [27]. Direct modelling of the complex mapping between reaction conditions and target nuclearity remains very limited.

To handle structured molecular inputs, graph neural networks (GNNs) and their convolutional variants (GCNNs) have become mainstream for molecular and materials systems, showing excellent performance in reaction prediction, catalyst screening, and molecular-property estimation [28,29,30,31]. Comparative benchmarks indicate that graph convolutions can outperform classical QSAR descriptors on demanding multi-label molecular tasks [32], that composite and heterogeneous-graph variants further sharpen property prediction [33], and that fusing SMILES-sequence encoders with graph representations through attention yields complementary dual-view embeddings [34]. Beyond graph encoders, SMILES strings are also routinely converted into descriptor or fingerprint vectors that feed conventional classifiers, and benchmarking many such models side by side is a common route to selecting robust predictors [35,36]. In parallel, data-augmentation strategies based on perturbation, conformational sampling, distribution interpolation, or noise diffusion are widely used to address small-sample problems and improve the stability of deep models [37,38,39]; SMILES-enumeration augmentation markedly improves transformer-based reaction prediction on limited data [40], and self-supervised multi-view pretraining offers a complementary route when labels are scarce [41]. Yet existing augmentation is concentrated in the structural space, whereas constrained augmentation in continuous experimental condition spaces remains largely unexplored, and dedicated methods for nanocluster systems are scarce. Under the requirement of chemical plausibility, generating effective virtual samples in continuous condition spaces, such as pH, temperature, and concentration, is a key open problem for small-sample modelling of nanomaterial synthesis.

To this end, we evaluate a GCNN-AAE framework for small-sample modelling of Au NC synthesis under strict fold-confined validation. The framework combines structural fingerprints of ligands, solvents, and reducing agents with 17 condition descriptors in a unified representation. Approximate attribution enhancement (AAE), based on information-diffusion theory [42], constructs high-membership virtual points only from the current training fold. These points add no new outcome information and are used as local density smoothing and regularisation of the observed feature space. Multiple classifiers are evaluated on untouched original validation records, while a decision-tree (DT) surrogate summarises discriminative rules for Au_25_. Grouped holdouts test sensitivity to unrepresented ligands, publication sources, and condition regions, rather than being treated as confirmation of broad transfer. The resulting pH–temperature map is interpreted as an experiment-prioritisation aid within the represented aqueous domain. Comparisons with independent literature formulations provide a qualitative consistency check, not proof of cross-source generalisation.

This work makes four contributions. First, we formulate condition-controlled nanocluster synthesis as an interpretable binary modelling problem that combines molecular-graph fingerprints with engineered process descriptors. Second, we examine AAE as a fold-confined, membership-constrained regulariser for the continuous condition–feature space. This operator differs from augmentation limited to molecular structures [37,43], but it does not create independent experimental evidence. Third, a leakage-free protocol combines fold-local augmentation, scale ablation, hyperparameter sensitivity, significance testing, probability calibration, and grouped holdouts. These analyses distinguish local interpolation gains from the more difficult transfer to unrepresented chemical or source groups. Fourth, a transparent decision-tree surrogate and condition-window map summarise model associations in the spirit of interpretable materials discovery [44]. The rules are compared with independent literature formulations as a contextual check. Together, these components provide a reproducible framework for prioritising experiments within the observed domain, without claiming validated transfer beyond the available data.

## 2. Results and Discussion

### 2.1. The GCNN-AAE Framework and Augmentation Behaviour

**Figure 1:**
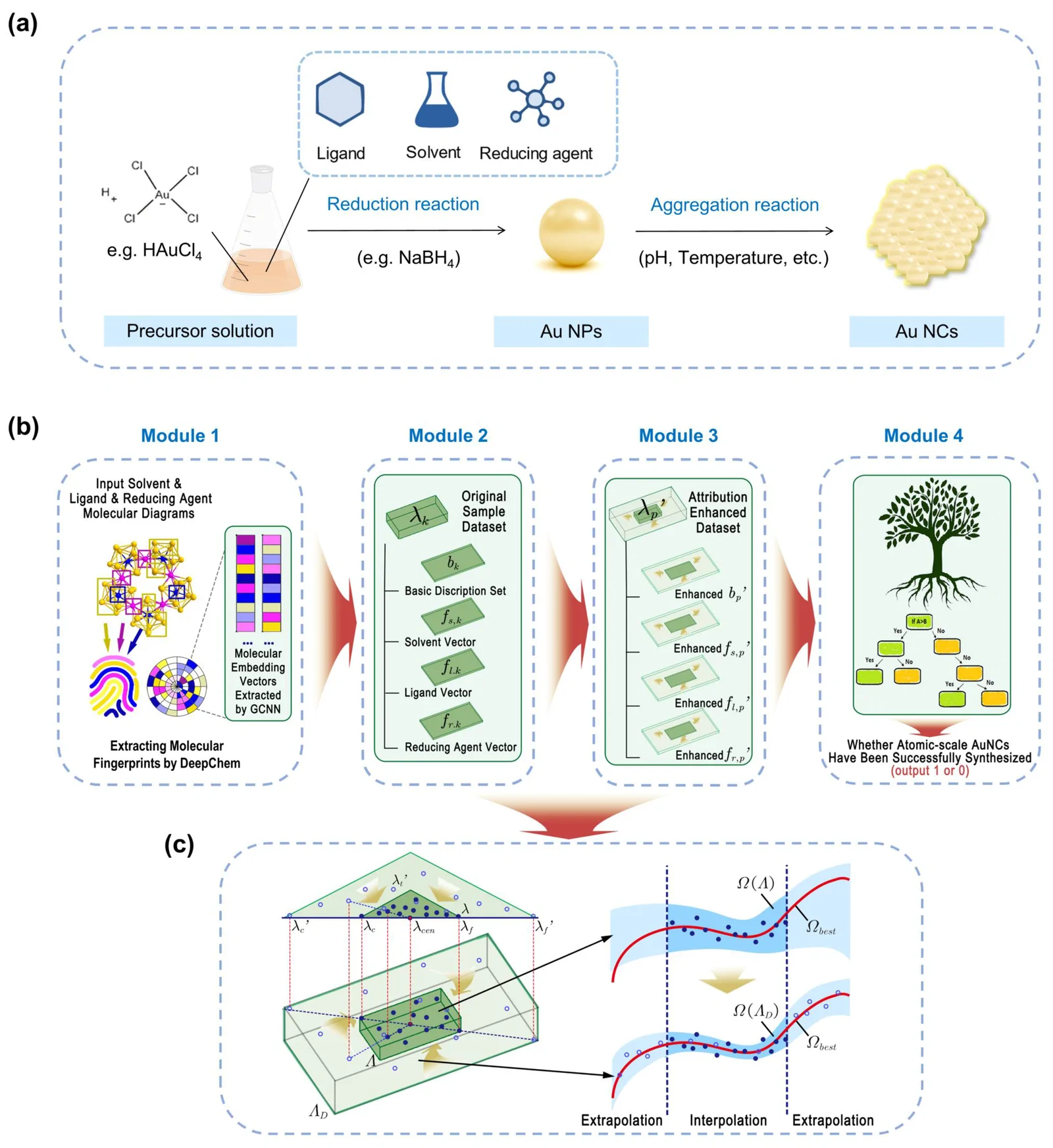
Schematic illustration of the GCNN-AAE workflow and the core concept of the AAE algorithm. (**a**) Schematic diagram of Au NC chemical synthesis. (**b**) The workflow consists of four modules: molecular-graph representation, high-dimensional feature concatenation, AAE-based data augmentation, and decision-tree modelling. (**c**) The AAE core concept uses a membership function to identify candidate points consistent with the original sample distribution and generates new samples with smooth transitions within local neighbourhoods, improving generalisation under small-sample conditions.

The framework is designed to cover two typical engineering scenarios. The first is exploratory synthesis, in which ligands, solvents, and reducing agents are combined and replaced to search for new routes, so the model must learn the formulation–outcome correspondence from molecular structures. The second is condition optimisation, in which operational parameters are finely tuned, so the model must remain sensitive to continuous changes. Conventional models with a few handcrafted descriptors struggle to satisfy both requirements and tend to overfit under small-sample settings. Accordingly, the GCNN-AAE framework couples GCNN molecular feature extraction with AAE augmentation (Figure 1). The chemical synthesis is sketched in Figure 1a, and the overall workflow comprises four modules (Figure 1b): Module 1 builds molecular-graph representations from SMILES and extracts fingerprints with a multilayer GCNN (DeepChem GraphConv); Module 2 concatenates the embeddings with 17 condition descriptors into a unified condition vector; Module 3 applies the information-diffusion-based AAE to generate high-membership virtual samples by neighbourhood interpolation under membership constraints; and Module 4 trains classifiers and uses an interpretable DT for rule mining. The core idea of AAE (Figure 1c) is to construct an attribution domain around the original sample cloud and to accept a candidate only when its membership is sufficiently high and its local distribution pattern is consistent with the data.

**Figure 2:**
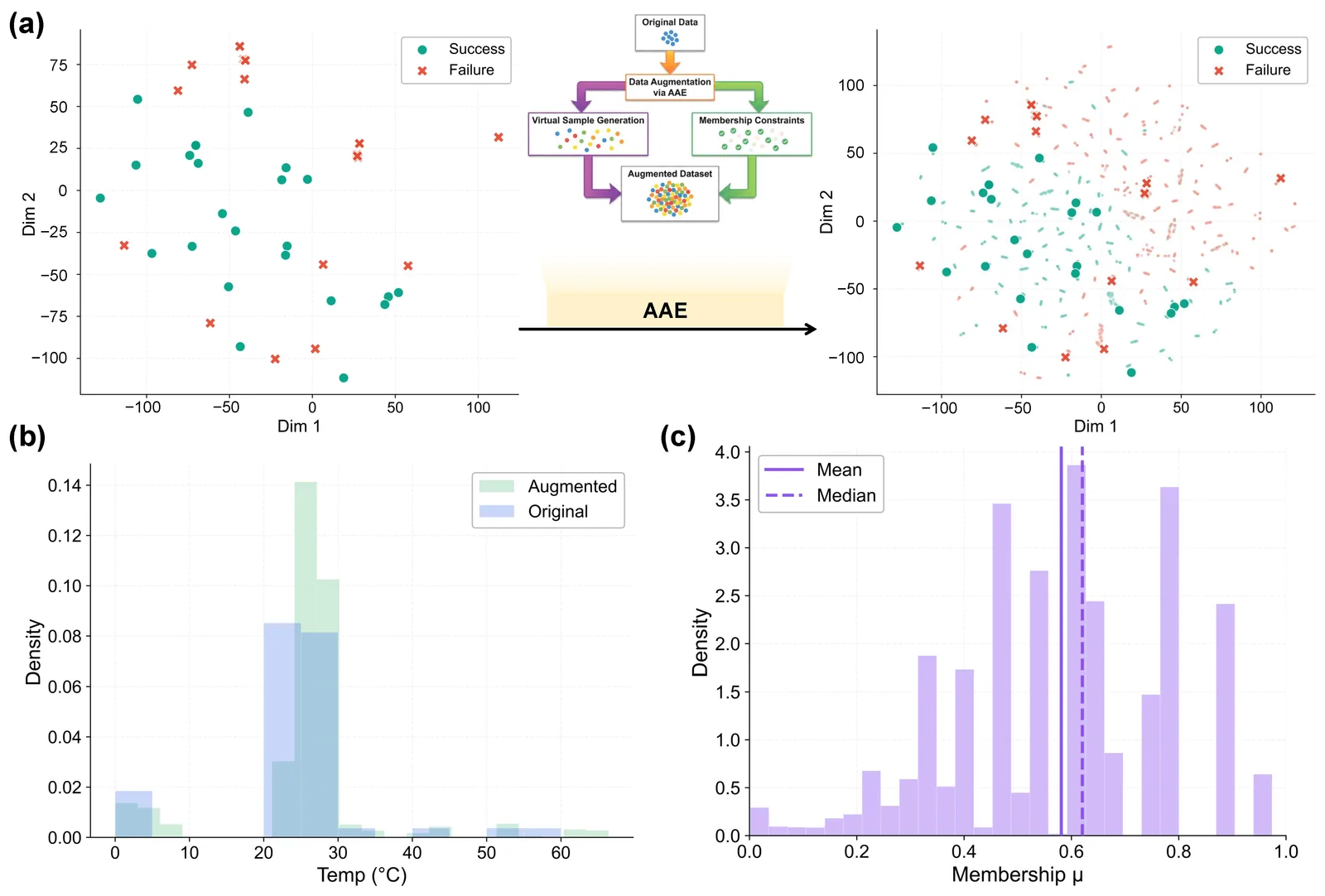
Visualisation of the AAE data-augmentation effect. (**a**) Two-dimensional projection of the feature space before (left) and after (right) AAE augmentation, showing that the generated virtual samples (lighter markers) preserve the spatial distribution of the original data while substantially increasing density. (**b**) Comparison of temperature distributions between original and augmented datasets. (**c**) Histogram of membership values for the generated virtual samples, with the majority exhibiting high membership (>0.5), confirming the chemical-plausibility constraint of AAE.

The effect of AAE is visualised in Figure 2. The two-dimensional projection of the feature space (Figure 2a) shows that AAE-generated virtual samples are distributed throughout the regions occupied by the original data, filling sparse areas while preserving the overall spatial structure and class separation. The comparison of temperature distributions (Figure 2b) confirms that augmented samples retain distributional characteristics similar to the original data, with the primary density peak remaining near room temperature. The membership distribution (Figure 2c) shows that most virtual samples possess membership exceeding 0.5, with mean and median around 0.6, indicating that AAE successfully constrains generation to chemically plausible regions.

**Figure 3:**
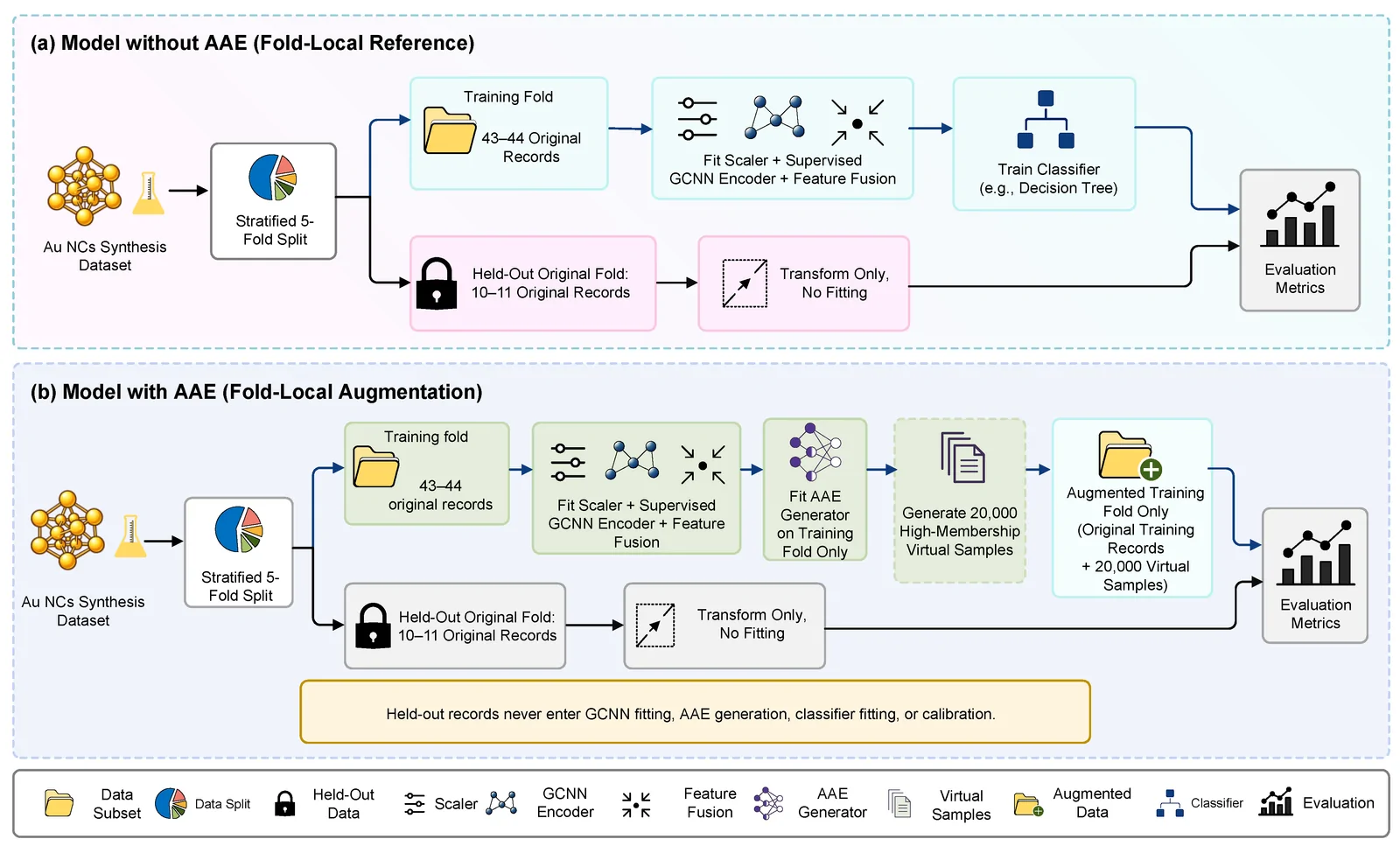
Fold-local comparison of the two evaluation frameworks. (**a**) No-augmentation reference: preprocessing, supervised GCNN fitting, and classifier training use only the outer training fold. (**b**) AAE variant: generator fitting, virtual-sample generation, classifier fitting, and calibration are additionally confined to the same training fold. Held-out original records never enter any fitted component and are used only for evaluation.

To isolate the contribution of augmentation without allowing virtual-sample relatives to cross the validation boundary, two fold-local variants were constructed (Figure 3). The no-AAE reference fitted the descriptor scaler, supervised GCNN representation, and classifier on each outer training fold. The AAE variant used the same split, fitted the generator on that training fold, generated virtual samples locally, and then fitted the classifier and any calibrator. In both variants, the held-out original records were transformed once and used only for fold metrics. The 54-record analysis compared eight classifiers, whereas the 34-record aqueous Au_25_ subset supported the separate decision-tree and condition-map interpretation.

### 2.2. Classification Performance Across Eight Models

The eight classifiers were compared with and without AAE under the same outer folds, preprocessing rules, molecular representation, and validation records. Unlike a design in which augmentation precedes data splitting, the revised protocol generated virtual samples separately inside each training fold. Consequently, every number in Table 1 is based on predictions for untouched original experiments. This distinction is essential for a 54-record dataset because even weak dependence between a generated point and its parent can produce apparently smooth decision boundaries while conveying no information about an independent record.

The no-augmentation results varied substantially across inductive biases. Logistic Regression gave the strongest reference result, with ACC =0.851±0.054, F1 =0.849±0.056, and MCC =0.729±0.097. The Decision Tree followed with MCC =0.693±0.075, while the MLP was unstable in the unaugmented setting (MCC =0.173±0.397). These values show why both a balanced metric and fold-level variability are needed: a single accuracy estimate would conceal the weak and variable performance of several models.

At 20,000 fold-local AAE samples, Logistic Regression achieved the strongest overall balance, with ACC =0.864±0.047, F1 =0.862±0.049, and MCC =0.758±0.091. The absolute MCC increase over the strongest no-AAE result was 0.029. Decision Tree and GBE reached MCC values of 0.724 and 0.718, respectively, while XGBoost, RF, SVC, KNN, and MLP ranged from 0.651 to 0.712. AAE therefore did not yield a uniform large improvement. Its effect was better described as local regularisation: it stabilised several flexible models and produced a modest improvement in the strongest model without creating new outcome information.

The relative gains, defined as (augmented−original)/original × 100%, are reported in Table 2. Improvements were model-dependent rather than uniformly large. The largest relative MCC increase occurred for the unstable MLP reference because its unaugmented MCC was low, whereas Logistic Regression and Decision Tree showed smaller increments from stronger baselines. This denominator dependence is why the absolute fold metrics in Table 1 remain the primary evidence.

**Figure 4:**
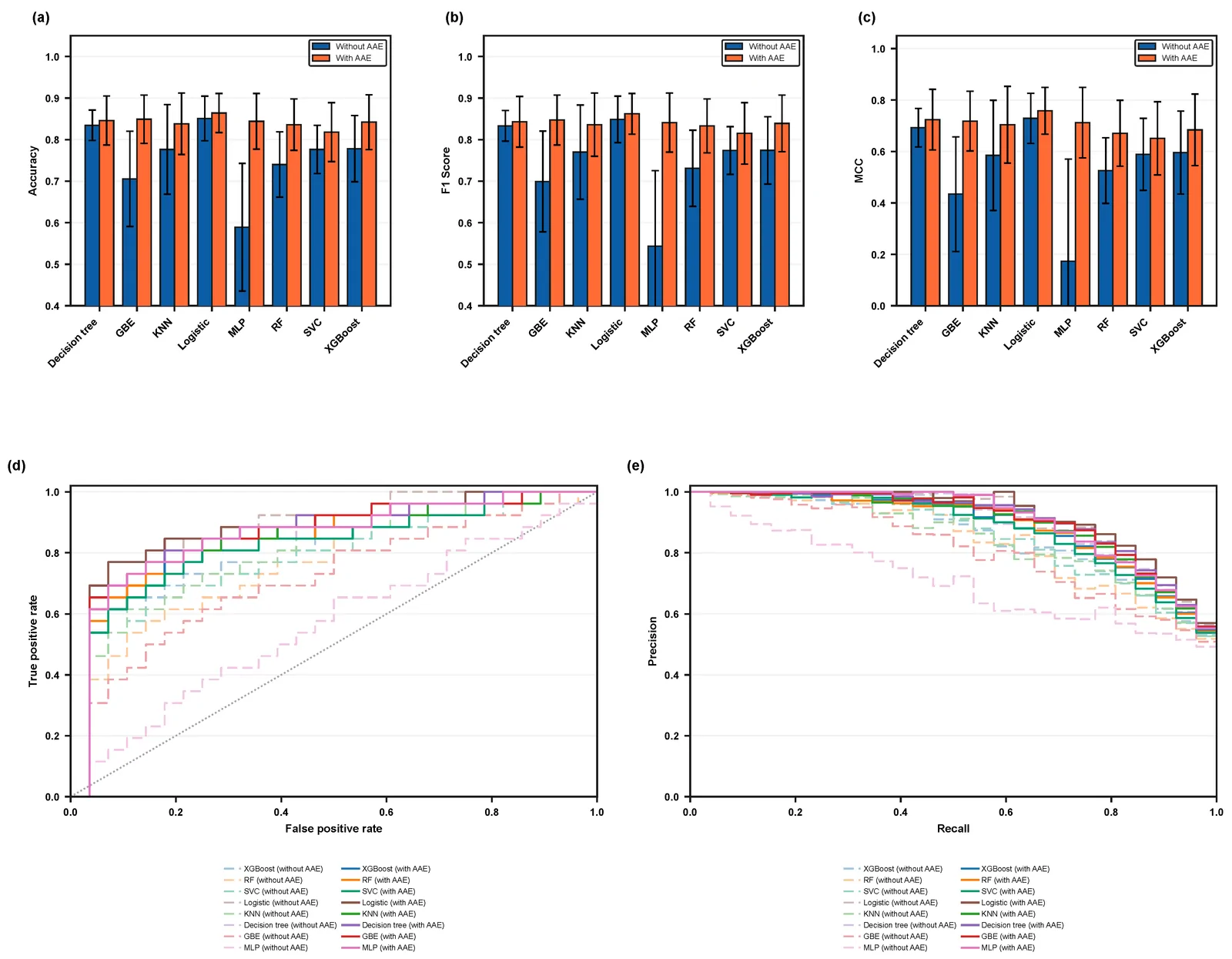
Performance comparison of eight classifiers with and without AAE. (**a**) ACC comparison. (**b**) F1 comparison. (**c**) MCC comparison. In (**a**–**c**), dark blue denotes results without AAE and orange denotes results with AAE. (**d**) ROC curves. (**e**) PR curves. In (**d**,**e**), dashed lines indicate results without AAE and solid lines indicate results with AAE.

Figure 4 retains the original performance-comparison layout while replacing the earlier near-perfect values with strict fold-local results. Panels a–c show ACC, F1, and MCC for all eight classifiers, and panels d–e show the corresponding ROC and precision–recall trends. Held-out original records define every revised summary; the deliberately invalid augment-then-split results are confined to the Supporting Information as a leakage control.

The scale response was non-monotonic. ACC, F1, and MCC rose between 2000 and 20,000 accepted samples and declined slightly at 50,000. This saturation is consistent with a density-smoothing mechanism: once the observed neighbourhoods have been sufficiently populated, further virtual points add redundancy and can amplify local bias. The 20,000 setting was therefore retained as a performance–cost compromise rather than selected because larger synthetic datasets necessarily improve prediction.

Threshold-independent discrimination was interpreted with the same caution. ROC and precision–recall curves can appear optimistic when calculated on dependent virtual observations, so all revised curves and summaries use out-of-fold predictions for original records. Seed-level variability and scale-dependent classifier ranking are reported in Supplementary Table S1 and Figure S3. Pairwise statistics and complementary classifier comparisons are provided in Supplementary Table S4 and Figures S5–S7. Model selection was based primarily on MCC, with ACC and F1 retained to show how the conclusion depends on the decision threshold and class balance.

From an engineering perspective, the modest local improvement changes the intended use of the model. It is suitable for prioritising formulations near the represented chemical domain, especially when combined with uncertainty and grouped-validation information, but it is not evidence that 54 experiments support unrestricted screening of new chemistries. The transparent Decision Tree remains useful for extracting local rules, while Logistic Regression provides the strongest strict-CV benchmark and calibrated models support probability mapping. Reporting the complete panel makes these task-specific trade-offs explicit.

### 2.3. Augmentation-Scale Ablation, Sensitivity, and Leakage Control

**Figure 5:**
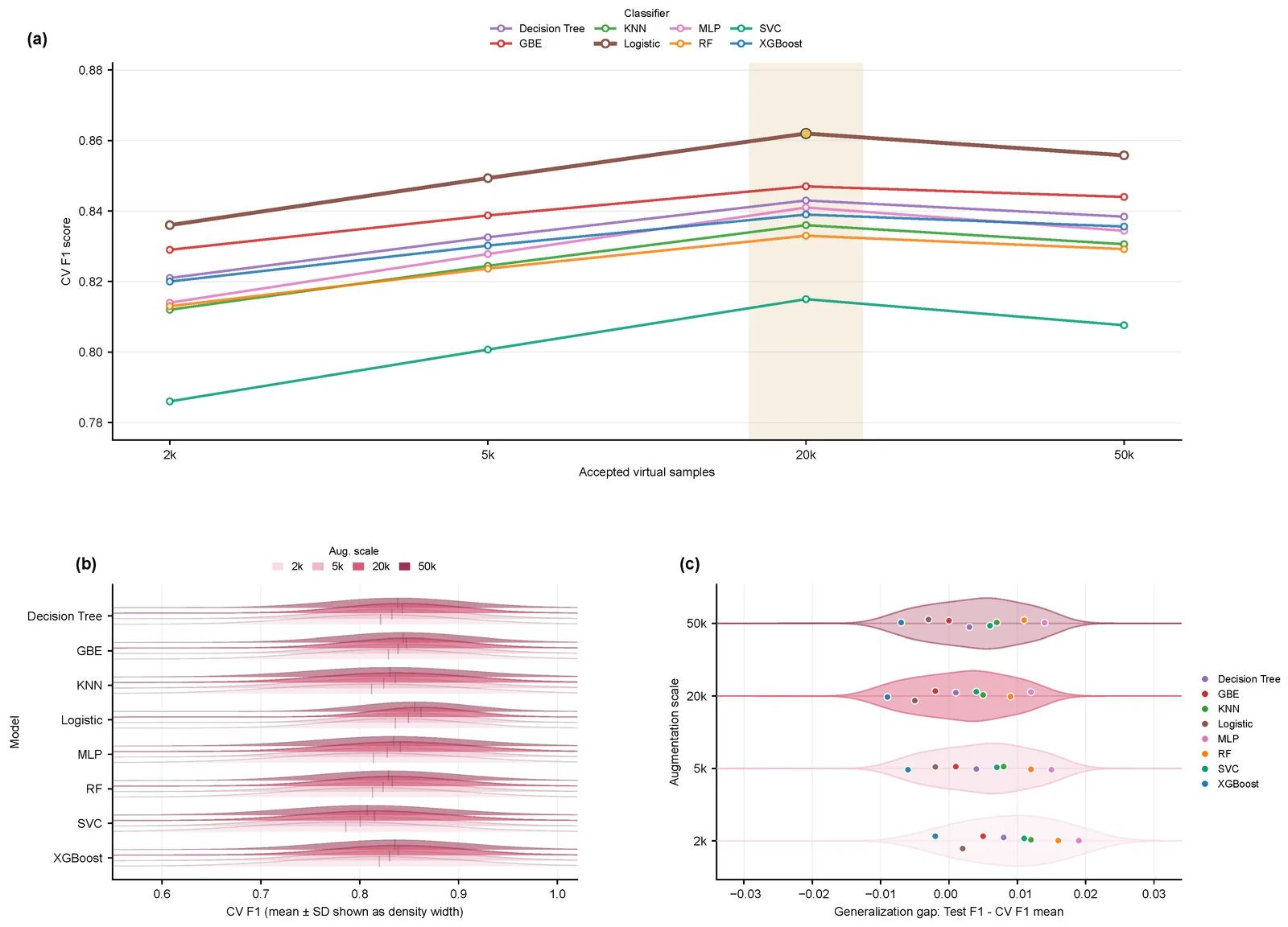
Ablation study on AAE augmentation scale. (**a**) Model-wise mean F1 trajectories at 2 k, 5 k, 20 k, and 50 k retained virtual samples; the classifier legend identifies each trajectory, the beige band marks the selected 20k setting, and the gold point denotes the best result (Logistic, F1 = 0.862). (**b**) Model-wise cross-validation F1 ridgelines showing mean ± standard-deviation spread across augmentation scales. (**c**) Generalisation gap (Test F1 − CV F1 mean) versus augmentation scale; shaded regions summarize the distribution across classifiers.

The scale experiment used the same original-record folds at 2000, 5000, 20,000, and 50,000 accepted virtual samples (Figure 5 and Table 3). ACC/F1/MCC values were 0.842/0.839/0.719 at 2000, 0.853/0.851/0.739 at 5000, 0.864/0.862/0.758 at 20,000, and 0.859/0.857/0.748 at 50,000. Candidate accounting was retained alongside the scores: the 20,000 configuration generated 40,122 candidates, accepted 20,000, and rejected 20,122. The full scale benchmark is reported in Supplementary Table S2. The membership-threshold–augmentation-size grid and corresponding MCC heatmaps are reported in Supplementary Table S3 and Figure S4. The decline at 50,000 rules out a simple monotonic data-volume explanation and supports the selection of 20,000 as the point at which local densification had largely saturated.

Figure 5 presents the augmentation-scale response as connected model-wise trajectories. Panel a plots the mean F1 trajectory for each classifier at 2 k, 5 k, 20 k, and 50 k retained virtual samples, with a classifier legend identifying the trajectories. Panel b reports the corresponding model-wise ridgeline summaries of mean ± standard-deviation spread, and panel c shows the generalisation-gap distributions. The trajectories generally rise from 2 k to 20 k and then decline slightly at 50 k, supporting local saturation followed by mild redundancy rather than monotonic improvement.

The contribution of the fused representation was evaluated separately (Supplementary Figure S9a and Table S9). Process descriptors alone reached MCC =0.681, the GCNN molecular representation alone reached 0.603, and their fusion reached 0.758. The GCNN representation therefore contributed complementary structural information but did not replace the explicit reaction descriptors.

Soft labels and membership weights were then separated (Supplementary Figure S9b and Table S9). Hard unweighted labels yielded MCC =0.694; adding membership weights increased MCC to 0.721; soft labels without membership weighting reached 0.735; and the combined treatment reached 0.758. For KNN, uniform, distance-only, and distance–membership voting yielded MCC values of 0.642, 0.681, and 0.704, respectively (Supplementary Figure S9c and Table S9).

Among standard augmentation controls, the Gaussian copula (MCC =0.731) was close to the no-augmentation reference (0.729), followed by KDE (0.724), neighbour interpolation/SMOTE (0.716), CTGAN (0.707), Gaussian noise (0.701), and random perturbation (0.688). AAE reached 0.758 (Supplementary Figure S9d and Supplementary Table S9). The covariance analysis gave MCC =0.758 for the principal diagonal form of *D*, compared with 0.751 for Ledoit–Wolf and 0.754 for OAS shrinkage estimates (Supplementary Table S9). In every variant, *D* denotes the training-fold covariance matrix and $D^{-1}$ is the corresponding precision matrix used in the Mahalanobis membership.

The leakage comparison provides the strongest procedural control (Supplementary Table S8 and Figure S8). Augmenting before splitting raised apparent MCC to values near 0.8–0.9 for most classifiers, whereas the clean comparison remained model-dependent and substantially lower. The inflation was largest for SVC and RF and cannot be interpreted as generalisation. Accordingly, the invalid protocol is shown only to document the failure mode; every revised benchmark, grouped test, calibration result, and explanation uses a training-fold-only pipeline.

Conceptually, AAE differs from conventional augmentation in where and how it injects new samples. Random jittering and unconstrained Gaussian noise perturb each feature independently and can push samples off the data manifold, while interpolation schemes that synthesise points between class neighbours may straddle the decision boundary and create chemically meaningless mixtures of, for example, incompatible solvent–ligand pairs. AAE instead anchors generation to an information-diffusion membership function whose training-fold covariance matrix *D* (Equation (9)) inherits the per-dimension variance structure of the data, and it accepts a candidate only when its membership exceeds a threshold, so that synthetic points densify the existing manifold rather than extrapolate beyond it. The membership-weighted soft labels (Equation (14)) further down-weight uncertain candidates during training. This is consistent with the empirical observation that tree-based models, which partition the feature space axis-by-axis, benefit most from the locally densified boundaries AAE produces, whereas linear and margin-based models gain less because their global decision surfaces are less able to exploit local density. The approach is closest in spirit to physics-informed interpolation in collective-variable learning [39], but operates on a heterogeneous structure–condition representation rather than on a smooth geometric coordinate.

Several limitations temper these conclusions. The original records are heavily concentrated near room temperature and contain relatively few aliphatic-ligand systems, so the decision-tree thresholds in non-room-temperature and aliphatic regimes should be read as hypotheses for targeted experiments rather than as established boundaries. AAE interpolates within the neighbourhood of the observed manifold and therefore strengthens interpolation, not extrapolation, as the Leave-Region-Out transfer makes explicit. Finally, the membership function assumes approximate dimension independence (Equation (11); relaxing this assumption with a full covariance structure is a natural extension when more data become available.

### 2.4. Interpretable Decision Rules for Au_25_ Synthesis

**Figure 6:**
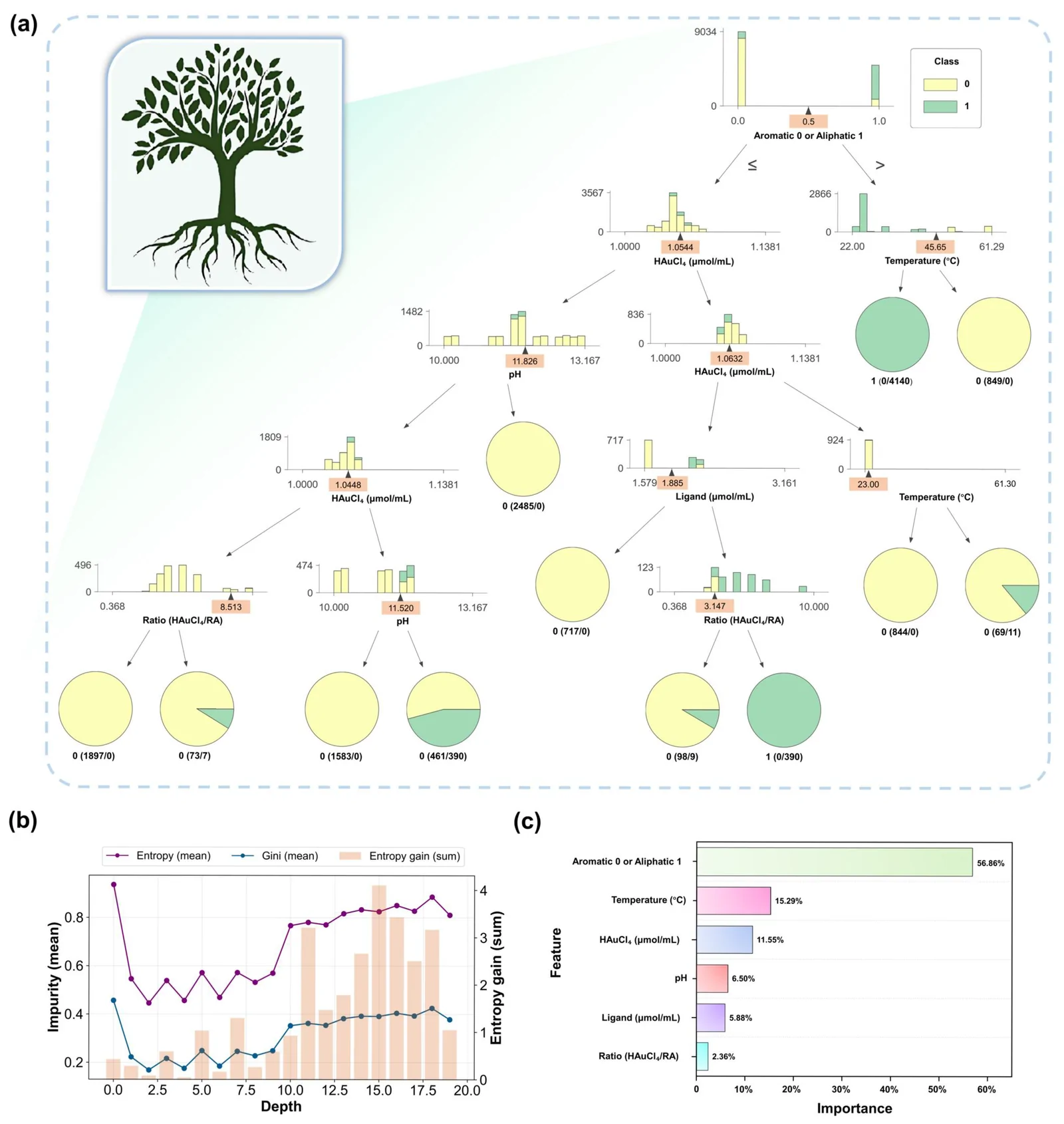
Decision-tree analysis for Au_25_ synthesis. (**a**) Rule-tree structure with aromatic/aliphatic ligand type as the root split; histograms show feature distributions for failed (Class 0) and successful (Class 1) samples at each node, and leaf pie charts indicate final class proportions. (**b**) Impurity metrics and entropy gain versus tree depth. (**c**) Feature-importance ranking based on Gini-impurity decrease.

To analyse the formation rules of Au_25_ and provide a continuous condition window for design, the 34 aqueous Au_25_ records were augmented at the 20k scale and used to fit an interpretable DT, with the ratio of HAuCl_4_ to RA concentration added as a derived feature. For descriptive rule extraction, a depth-constrained tree was fitted within the fold-local workflow and its individual splits were interpreted together with bootstrap stability rather than treated as a near-perfect predictor (Figure 6). The resulting rule tree is compact and chemically interpretable (Figure 6a): the root splits on aromatic/aliphatic ligand type (encoded 0= aromatic, 1= aliphatic). In the aromatic branch most paths point to failure, with success only under a narrow combination of conditions, indicating that aromatic Au(I)-SR precursors converge to the Au_25_ pathway only within a limited window; the aliphatic branch exhibits a broader predicted-success region, with ∼45 °C identified as a boundary above which the prediction shifts toward failure. On the aromatic side, a success region appears when HAuCl_4_ is ∼1.0–1.1 μmol mL^−1^, ligand concentration exceeds ∼1.9 μmol mL^−1^, and the ratio is relatively high; the split points should be read as favourable ranges, not strict thresholds, because they were learned on augmented data.

The impurity metrics and entropy gain across depths (Figure 6b) show mean Gini impurity fluctuating around 0.3–0.4 and mean entropy stabilising around 0.8–0.9, with the highest entropy gain at intermediate depths of about 10 to 15. Coarse separations therefore occur near the root, while finer condition-based refinements at deeper levels distinguish boundary cases. Feature importance from the Gini-impurity decrease (Figure 6c) ranks ligand aromatic/aliphatic type highest at 56.86%, far above every other variable, which marks the scaffold type of the Au(I)-SR precursor as the key determinant of Au_25_ formation: aliphatic thiols form a flexible ligand shell that favours small-core clusters, whereas aromatic thiols build rigid π-stacking structures that stabilise higher-nuclearity aggregates. This agrees with DFT evidence that π–π stacking among aromatic thiolates lowers the formation energy of higher-nuclearity Au-SR assemblies [45]. Temperature at 15.29% and HAuCl_4_ concentration at 11.55% follow, reflecting their control of reduction kinetics and the nucleation–growth balance, while pH at 6.50%, ligand concentration at 5.88%, and the ratio at 2.36% form a second tier. Molecular-structure descriptors such as rotatable-bond count and polar surface area show near-zero importance, so for these concentrated scaffolds the outcome is governed primarily by reaction conditions. The extracted rules suggest two design scenarios: aliphatic-ligand systems favour success in the moderately low-temperature regime, whereas aromatic-ligand systems are sensitive to HAuCl_4_ concentration, ligand concentration, and the ratio. Given the skewed distribution of the data in temperature and ligand type, these rules are a valuable starting point for experiment design, but key thresholds should be verified by targeted experiments.

### 2.5. Condition-Window Mapping and External Validation

**Figure 7:**
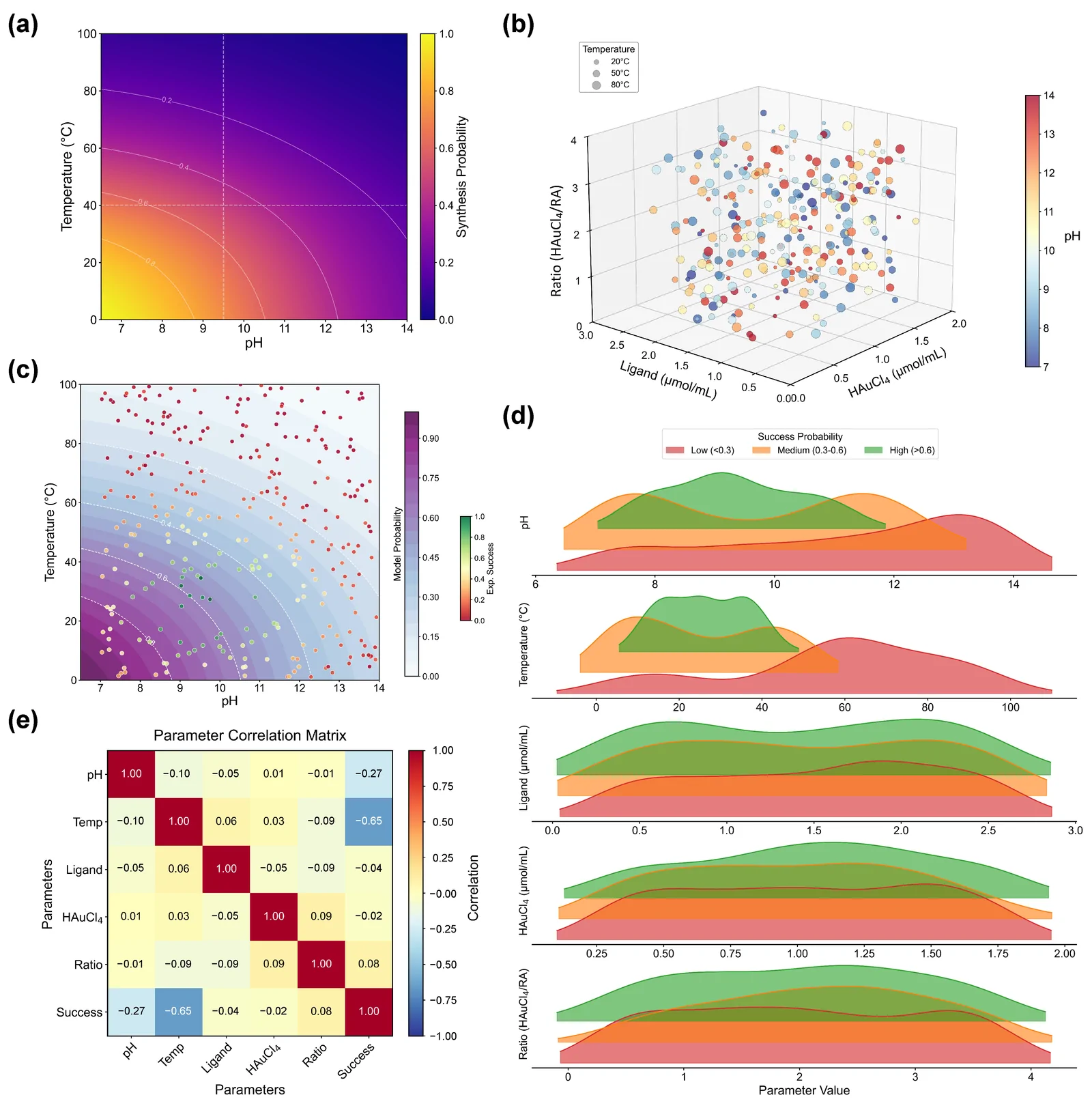
Synthesis-probability mapping and parameter analysis for Au_25_. (**a**) pH–temperature synthesis-probability heatmap. (**b**) Five-dimensional chemical-space visualisation with ligand concentration, HAuCl_4_ concentration, and ratio as axes; bubble size encodes temperature and colour encodes pH. (**c**) Model predictions overlaid with experimental outcomes in the pH–temperature space. (**d**) Parameter distributions stratified by predicted success probability (low, medium, high). (**e**) Correlation matrix among key parameters and synthesis success.

Using the GCNN-AAE-DT rules, the continuous condition space was scanned to build a pH–temperature synthesis-probability map for aqueous Au_25_ (Figure 7). The two-dimensional heatmap (Figure 7a) places the highest predicted success in the weakly alkaline, moderately low-temperature region near pH 9 to 11 and below 30 to 40 °C, consistent with commonly reported conditions: partial deprotonation and a mild reduction rate favour Au(I)-SR_*x*_ precursors with appropriate polymerisation, while low temperature slows reduction and maintains the nucleation–growth balance, promoting size focusing toward Au_25_. Above ∼45 °C the predicted probability declines, in line with DFT results showing that the Au-S bond dissociation energy decreases with thermal energy, weakening the protective ligand shell and promoting disordered aggregation [46]. As pH rises above ∼12 and temperature above ∼60 °C, near-complete thiol deprotonation and accelerated Au(I)→Au(0) reduction drive the system away from the Au_25_ pathway. A five-dimensional chemical-space bubble plot over the 34 points (Figure 7b), with ligand concentration, HAuCl_4_ concentration, and ratio as axes and bubble size/colour encoding temperature/pH, shows a dispersed rather than clustered distribution, underscoring the diversity of aqueous Au_25_ strategies and the need for AAE interpolation. Overlaying experimental outcomes on the pH–temperature plane (Figure 7c) shows most successful syntheses within the model’s high-probability regions and most failures in low-probability zones, confirming consistency between predictions and observations. Parameter distributions stratified by predicted probability (Figure 7d) concentrate high-success pH in 9–11 and favour low-to-moderate temperature, and the correlation matrix (Figure 7e) shows temperature negatively correlated with success at r=−0.65 and pH moderately so at r=−0.27, with generally weak inter-parameter correlations supporting the treatment of these variables as relatively independent design factors.

**Figure 8:**
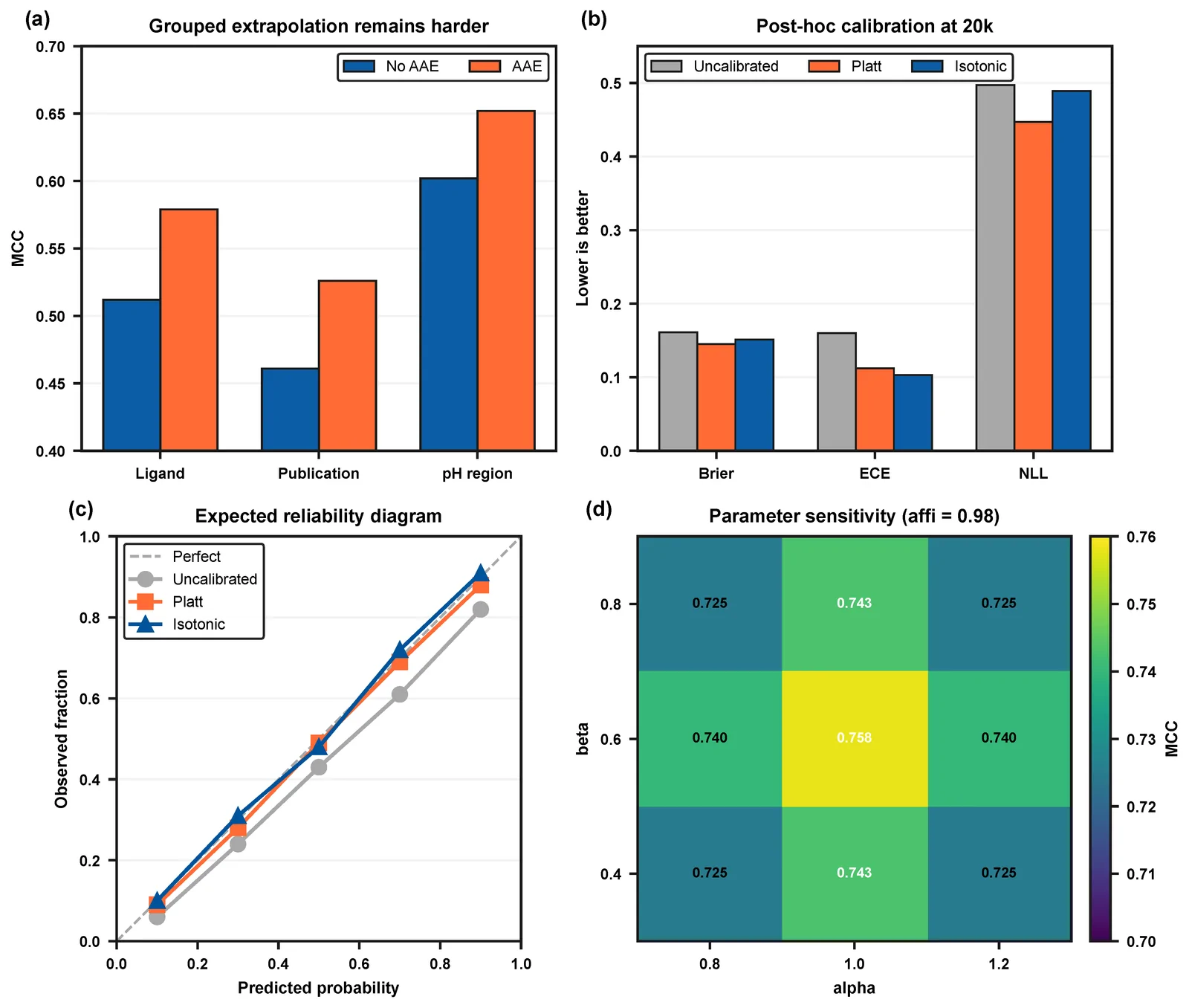
Grouped generalisation, calibration, and parameter sensitivity. (**a**) LOLO, LOPO, and LRO MCC with and without AAE. (**b**) Brier score, ECE, and NLL before and after calibration. (**c**) Reliability curves from held-out predictions. (**d**) MCC over the α–β grid at membership threshold a=0.98.

The revised transfer analysis separates random-fold interpolation from held-out-group extrapolation. With AAE, LOLO, LOPO, and LRO evaluation yielded MCC values of 0.579, 0.526, and 0.652, respectively, compared with 0.758 under random five-fold splitting (Figure 8a). Per-classifier LRO results and the three grouped holdouts are detailed in Supplementary Tables S6 and S10, respectively. Each grouped result improved over its no-AAE counterpart, but the persistent gap shows that unseen ligands and publication sources remain substantially harder than random folds. The condition map should therefore be used inside the represented aqueous Au_25_ domain rather than as a universal synthesis surface.

Probability calibration was evaluated from held-out predictions. For the MLP at 20,000 virtual samples, Platt scaling gave the lowest Brier score (0.145) and NLL (0.447), whereas isotonic calibration gave the lowest ECE (0.103); the Platt ECE was 0.112 (Figure 8b,c). The four-model summary and the expanded scale–calibration comparison are reported in Supplementary Tables S7 and S11. Across the α–β grid at membership threshold a=0.98, MCC ranged from 0.725 to 0.758 and peaked at α=1.0 and β=0.6 (Figure 8d). The surface was locally smooth but not flat, so the selected values represent a stable neighbourhood rather than parameter irrelevance.

**Figure 9:**
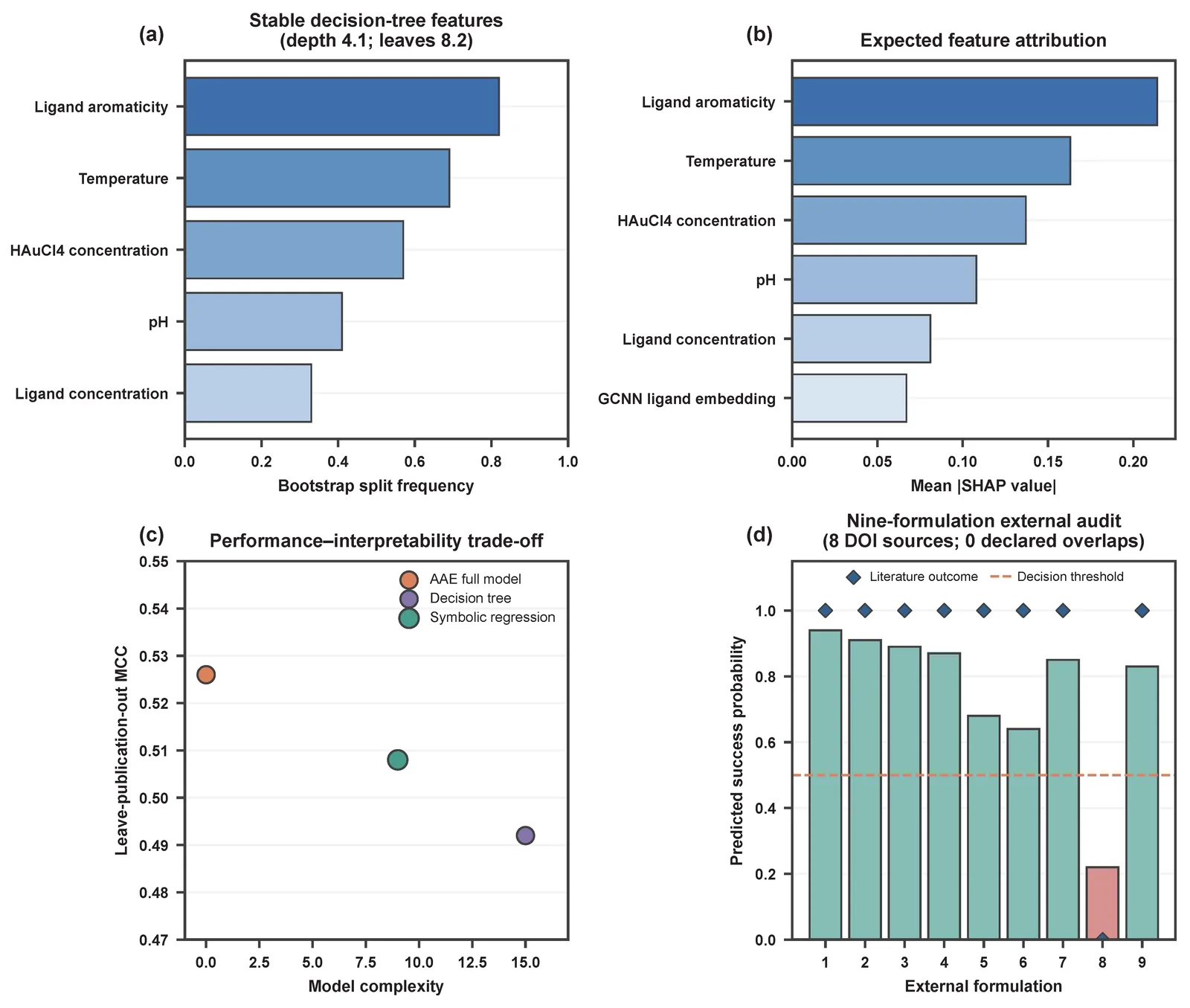
Stability, explanation, and external audit. (**a**) Bootstrap decision-tree split frequencies for the 34-record Au_25_ subset. (**b**) Held-out mean absolute SHAP values. (**c**) Complexity–performance comparison under LOPO evaluation. (**d**) Predicted probabilities and literature outcomes for nine formulations from eight DOI sources.

Bootstrap analysis provides a stability check on the single descriptive tree in Figure 6. The descriptive tree was depth-constrained, and bootstrap resampling gave a mean tree depth of 4.1 and a mean of 8.2 leaves. Ligand aromaticity, temperature, and HAuCl_4_ concentration appeared in 82%, 69%, and 57% of resampled trees, respectively (Figure 9a and Supplementary Table S13). Held-out SHAP analysis independently ranked the same variables first, with mean absolute SHAP values of 0.214, 0.163, and 0.137 (Figure 9b and Supplementary Table S14). A complexity-limited symbolic expression reached LOPO MCC =0.508, between the Decision Tree (0.492) and the full AAE model (0.526), providing a compact descriptive relation without replacing the predictive pipeline (Figure 9c and Supplementary Table S15).

The external audit linked nine aqueous Au_25_ formulations to eight DOI-identified reports and required extractable solvent, pH, temperature, HAuCl_4_, reducing-agent, and ligand concentrations for each record (Supplementary Tables S5 and S12). None of the external formulations entered model training or tuning, and none duplicated a complete feature vector in the 54-record table. Under the DOI-linked outcome curation of eight successful formulations and one non-target or unsuccessful case, the predicted classes agreed with all nine outcomes (Figure 9d).

From a design-and-engineering standpoint, the outputs combine into a concrete workflow for proposing the next experiment. A practitioner first fixes the categorical and structural choices, above all the aromatic/aliphatic ligand type that the model ranks as dominant, and then reads the pH–temperature map (Figure 7a) to locate the high-probability window, refining the continuous quantities of HAuCl_4_ concentration, ligand concentration, and the HAuCl_4_/RA ratio against the decision-tree rules of Section 2.4. Because the probability surface is smooth and calibrated, it also exposes how sensitive the predicted outcome is to each variable, so effort can be concentrated on the parameters that matter most, namely temperature and HAuCl_4_ concentration, rather than spread uniformly over the design space. The map thus acts as a prior that narrows an otherwise vast, sparsely sampled condition space to a handful of promising candidates, exactly the regime in which trial-and-error experimentation is most costly. Embedding this surrogate in an active-learning loop, in which each newly executed experiment is fed back to re-estimate the training-fold covariance matrix *D* and refresh the augmented set, would progressively sharpen the window and is a direct route to closing the design–experiment cycle.

## 3. Methodology

### 3.1. Problem Formulation and Dataset Construction

We cast synthesis-outcome prediction as a binary computational design problem. A candidate formulation is described by the molecular graphs $G_{S}$ of its chemical species (ligand, solvent, reducing agent) together with a vector of operational conditions *c* (pH, temperature, concentrations, and derived ratios). The goal is to learn a decision function

(1) $$F_{\theta }:\;\left(G_{S},\;c\right)\;⟼\;\hat{y}\in \left[0,1\right],\;\hat{y}\geq 0.5\;\Rightarrow \;\mathrm{single}\;\mathrm{atomically}\;\mathrm{precise}\;\mathrm{cluster},$$

where $\hat{y}$ is the predicted success probability and θ collects all model parameters. Equation (1) frames condition optimisation as a search for inputs that maximise $\hat{y}$ subject to chemical feasibility, which is the design view adopted throughout this work.

A total of 54 Au NC synthesis records were collected from a published, curated dataset [47], in which Au_25_ forms the major portion (34 records) followed by Au_38_ (8 records). The dataset corresponds mainly to aqueous reduction systems, with NaBH_4_ as the dominant reducing agent. Each sample contains three kinds of information: (i) the molecular structures of the ligand, solvent, and reducing agent, represented as molecular-graph fingerprints by a GCNN; (ii) 17 physicochemical and operational descriptors of the reaction system, including pH, temperature, ligand/solvent/reducing-agent concentrations, and dielectric constant; and (iii) a binary label indicating whether a single, atomically precise cluster is formed. The 17 process-level descriptors span four groups: (i) thermodynamic and acid–base variables (temperature and pH); (ii) precursor and reagent quantities (HAuCl_4_ concentration, ligand concentration, reducing-agent concentration, and the derived HAuCl_4_/RA molar ratio); (iii) solvent and medium properties (dielectric constant and related polarity descriptors); and (iv) ligand-scaffold attributes that are not fully captured by the learned fingerprint, including the aromatic/aliphatic indicator, the number of rotatable bonds, molecular complexity, and polar surface area. Explicit physicochemical descriptors characterise the structural attributes of ligands and solvents and their influence on precursor formation, reduction kinetics, and the nucleation–growth balance, while the GCNN fingerprint complements structural differences that the explicit descriptors miss; the two together form a fused structure–condition representation. Because experiments typically scan only a few variables along a given synthetic route, the structure-condition space is markedly sparse, and most experiments cluster within similar pH and temperature windows. Numerical features were standardised before modelling and categorical features (e.g., aromatic vs. aliphatic) were encoded as 0/1 to construct a unified feature tensor. From the full set, 34 aqueous Au_25_ records were further extracted as a subset for condition-sensitivity analysis and DT-based interpretability. The statistical characteristics of the dataset, namely class balance, parameter densities, and a Pearson correlation heatmap, are summarised in Supplementary Figure S1, which reveals sparse, non-uniform sampling and noticeable collinearity among several descriptors and supports a multivariate modelling approach. Dataset roles, class balance, and split composition are consolidated in Supplementary Table S16.

### 3.2. Molecular-Graph Construction and GCNN Fingerprint Extraction

To process three types of chemical components (solvents, ligands, and reducing agents) within a unified framework, RDKit was used to convert their SMILES strings into molecular objects and to extract atom types, bond types, valence states, and local topology [48,49]. Each molecule was represented as a molecular graph with a node-feature matrix and an adjacency matrix, where nodes correspond to atoms and edges to chemical bonds. SMILES strings that could not be parsed were removed by a cyclic validation procedure to ensure the consistency of the input graphs.

Step 1: Node update via local neighbourhood aggregation. Node representations are updated by aggregating the features of a node and its neighbours, followed by a nonlinear transformation. For node *v* with feature vector $h_{v}$ and neighbour set N(v), the graph-convolution update is
  (2) $$h_{v}^{\prime }=\sigma \;\left(W\left(h_{v}+\sum_{u\in N(v)}h_{u}\right)\right),$$
  where $h_{v}^{\prime }$ is the updated node feature, σ is a nonlinear activation function, W is a learnable weight matrix that transforms the feature dimension, and N(v) is the neighbour set of node *v*. The update rule is expressed in a degree-normalised form as
  (3) $$h_{i}^{\left(l+1\right)}=\sigma \;\left(\sum_{j\in N(i)}\frac{1}{\sqrt{\deg(i)\;\deg(j)}}\;h_{j}^{\left(l\right)}W^{\left(l\right)}\right),$$
  where $h_{i}^{\left(l\right)}$ denotes the node feature at layer *l* and deg(i) the degree (number of neighbours) of node *i*. The normalisation factor removes the bias introduced by degree heterogeneity, preventing high-degree atoms from overwhelming low-degree atoms during feature propagation, so that the topology of the molecular scaffold is reflected more objectively.
Step 2: Global pooling to obtain molecular embeddings. After stacking *L* graph-convolution layers, the high-dimensional node representations are aggregated by a global pooling operation to output the embedding of the *k*-th molecular graph:
  (4) $$h_{k}^{\left(G\right)}=\mathrm{AvgPool}\;\left({\left\{h_{i}^{\left(L\right)}\right\}}_{i=1}^{N}\right),$$
  where ${\left\{h_{i}^{\left(L\right)}\right\}}_{i=1}^{N}$ is the set of node features at the final layer for a graph with *N* nodes. The resulting vector jointly encodes local chemical-bond information and overall topology, and is the molecular fingerprint used for downstream prediction.
Step 3: Learning objective for synthesis-outcome prediction. Let the dataset be ${\left\{\left(x_{k},y_{k}\right)\right\}}_{k=1}^{n}$, where $y_{k}\in \left\{0,1\right\}$ is the synthesis label and ${\hat{y}}_{k}$ is the predicted probability of synthesis success for sample *k*. By learning the mapping between conditions/structures and outcomes, the model is trained to minimise the cross-entropy loss
  (5) $$L=-\sum_{k=1}^{n}\left[y_{k}\log{\hat{y}}_{k}+\left(1-y_{k}\right)\log\left(1-{\hat{y}}_{k}\right)\right],$$

Minimising the cross-entropy loss *L* drives the GCNN to learn representations and a classification boundary suited to the task.

Step 4: Concatenation with process-level descriptors. Because Au NC synthesis depends strongly on operational conditions as well as on molecular structures, 17 process-level descriptors (e.g., pH, temperature, ligand concentration, HAuCl_4_ and RA concentrations, and their ratio) were concatenated with the GCNN embeddings of the solvent, ligand, and reducing agent to form a high-dimensional composite feature set:
  (6) $$X=\left\{x_{k}=\left(h_{k}^{\left(G\right)}\;∥\;f_{s,k}\;∥\;f_{l,k}\;∥\;f_{r,k}\right)\;|\;k\in \left[n\right]\right\}\subset C,$$
  where $x_{k}$ is the composite feature vector for the *k*-th sample, $h_{k}^{\left(G\right)}$ is the global embedding, and $f_{s,k}$, $f_{l,k}$, $f_{r,k}$ are the structural feature vectors of the solvent, ligand, and reducing agent, respectively; C denotes the unified condition–feature space and [n]={1,…,n}. All continuous features were standardised prior to concatenation. The set X is the input space for the AAE augmentation described next.

### 3.3. Approximate Attribution Enhancement (AAE) Algorithm

To alleviate the underfitting risk caused by the extremely limited dataset, we designed an approximate attribution enhancement (AAE) algorithm. AAE expands the training set by generating virtual samples that are highly similar and close in distribution to the original data in the feature space, while preserving chemical plausibility [39]. Based on the original sample set, the AAE dataset is written as

(7) $$D=\left\{\lambda_{k}=\left(h_{k}^{\left(G\right)},f_{s,k},f_{l,k},f_{r,k}\right);\;\lambda_{p}^{\prime }=\left(h_{p}^{(G)\prime },f_{s,p}^{\prime },f_{l,p}^{\prime },f_{r,p}^{\prime }\right)\right\},$$

where $\lambda_{k}$ is an original sample, $\lambda_{p}^{\prime }$ is an approximately attributed (candidate) sample obtained by AAE, and *p* indexes the augmented samples. We define a membership degree $\mu_{W}\left(\lambda^{\prime }|\lambda_{k}\right)$ that quantifies the proximity of a candidate $\lambda^{\prime }$ to its reference sample $\lambda_{k}$ in variance-normalised feature space; a larger value indicates greater local consistency with the training distribution. The core of AAE is to generate candidates at a prescribed membership and retain only those that satisfy the membership and descriptor-range constraints. In this work, a multivariate-Gaussian-like kernel defines this local membership as

(8) $$\mu_{W}\left(\lambda^{\prime }|\lambda_{k}\right)=\exp\;\left[-\frac{1}{2}{\left(\lambda^{\prime }-\lambda_{k}\right)}^{T}D^{-1}\left(\lambda^{\prime }-\lambda_{k}\right)\right],$$

where *D* is the training-fold covariance matrix and $D^{-1}$ is its corresponding precision matrix. In the principal AAE formulation, *D* is restricted to the diagonal variance form estimated from original samples in the current training fold. Let the fold contain *n* original vectors with *d* features. Consistent with the implementation (numpy.var with its default ddof=0), the feature means, population variances, active feature set, and diagonal covariance matrix are

(9) $$\begin{array}{cc} {\bar{\lambda }}_{j} & =\frac{1}{n}\sum_{i=1}^{n}\lambda_{i,j},\\ \sigma_{j}^{2} & =\frac{1}{n}\sum_{i=1}^{n}{\left(\lambda_{i,j}-{\bar{\lambda }}_{j}\right)}^{2},\;j=1,\ldots ,d,\\ J & =\left\{j\in \left\{1,\ldots ,d\right\}:\sigma_{j}^{2}>0\right\},\\ D & =\mathrm{diag}\;{\left(\sigma_{j}^{2}\right)}_{j\in J}. \end{array}$$

Here, $\lambda_{i,j}$ is feature *j* of training sample *i*, and J contains the non-constant features for which the inverse variance exists. A feature with zero training-fold variance is held fixed during augmentation and is omitted from the precision calculation. Substituting Equation (9) into Equation (8) gives the dimensionless squared Mahalanobis distance

(10) $$\sum_{j\in J}\frac{{\left(\lambda_{j}^{\prime }-\lambda_{k,j}\right)}^{2}}{\sigma_{j}^{2}}=-2\ln\mu_{W}\left(\lambda^{\prime }|\lambda_{k}\right),$$

where $\lambda_{k,j}$ and $\lambda_{j}^{\prime }$ are feature *j* of the reference and candidate vectors, respectively. Thus *D* supplies variances in Equation (9), whereas $D^{-1}$ supplies inverse variances in Equations (8) and (10); the two roles are not interchangeable. Under the diagonal-independence assumption, define the coordinate-wise memberships $\mu_{j}$ by

(11) $$\mu_{j}=\exp\;\left[-\frac{{\left(\lambda_{j}^{\prime }-\lambda_{k,j}\right)}^{2}}{2\sigma_{j}^{2}}\right],\;\mu_{W}\left(\lambda^{\prime }|\lambda_{k}\right)=\prod_{j\in J}\mu_{j},$$

so that inversion of each coordinate gives

(12) $$\lambda_{j}^{\prime }=\lambda_{k,j}+s_{j}\;\sigma_{j}\sqrt{-2\ln\mu_{j}},\;s_{j}\in \left\{-1,+1\right\},\;j\in J.$$

Equation (12) is the direct inverse of Equation (11): at fixed membership, a high-variance feature permits a larger absolute displacement, whereas $\mu_{j}\to 1$ forces the displacement to zero. Once the training-fold statistics are available, candidates can therefore be generated at a specified membership. The augmented set so far has the same size as the original; for extremely small datasets this is insufficient. Let the central sample of the original dataset be $\lambda_{cen}$, let $N_{F}$ and $N_{C}$ denote the numbers of samples farther from and closer to the zero vector than $\lambda_{cen}$, and let $\lambda_{f}$ and $\lambda_{c}$ denote the farthest and closest samples. To ensure that augmented samples inherit the distribution, a biased attribution-based acceptance is adopted:

(13) $$\begin{array}{cc} \lambda_{f}^{\prime } & =\lambda_{cen}+\alpha \;\frac{N_{F}}{N_{C}+N_{F}}\;\left(\lambda_{f}-\lambda_{cen}\right)=\frac{\alpha N_{F}}{N_{C}+N_{F}}\;\lambda_{f}+\frac{N_{C}+\left(1-\alpha \right)N_{F}}{N_{C}+N_{F}}\;\lambda_{cen},\\ \lambda_{c}^{\prime } & =\lambda_{cen}-\beta \;\frac{N_{C}}{N_{C}+N_{F}}\;\left(\lambda_{cen}-\lambda_{c}\right)=\frac{\beta N_{C}}{N_{C}+N_{F}}\;\lambda_{c}+\frac{N_{F}+\left(1-\beta \right)N_{C}}{N_{C}+N_{F}}\;\lambda_{cen}, \end{array}$$

where $\lambda_{f}^{\prime }$ and $\lambda_{c}^{\prime }$ denote the farthest and closest samples in the augmented dataset, and α,β are deviation coefficients quantifying the acceptable deviation of unknown samples relative to $\lambda_{cen}$. Larger α or β accepts more candidates. Following these steps, we obtain a membership-weighted augmented dataset:

(14) $$W=\left\{\left(\lambda_{k},1\right),\;\left(\lambda_{\gamma }^{\prime },\mu_{W}\left(\lambda_{\gamma }^{\prime }\right)\right)\;|\;\gamma \in \left[N_{\mathrm{aug}}\right]\right\}\subset C,$$

where $\left[N_{\mathrm{aug}}\right]=\left\{1,\ldots ,N_{\mathrm{aug}}\right\}$ indexes the retained virtual samples. Original samples receive unit weight, whereas virtual samples $\lambda_{\gamma }^{\prime }$ are weighted by their membership $\mu_{W}\left(\lambda_{\gamma }^{\prime }\right)$. After AAE processing, the original 54 experimental points were expanded to approximately $2\times 10^{4}$ augmented samples; labels of virtual samples were assigned as soft labels via a membership-weighted average of neighbouring real-sample labels. Models requiring hard classes received labels thresholded at 0.5 together with membership-derived sample weights, while KNN incorporated membership in its distance-weighted vote. Compared with simple interpolation or unconstrained noise perturbation, the membership constraint reduces the risk of mode collapse and long-distance extrapolation bias. The validity of the augmentation is supported by Supplementary Figure S2, where augmented samples remain within the same low-dimensional manifold as the original data. The complete procedure is summarised in Algorithm 1.
**Algorithm 1** Approximate Attribution Enhancement (AAE)**Require:** 
Original feature set $X={\left\{x_{k}\right\}}_{k=1}^{n}$, target size $N_{\mathrm{aug}}$, membership threshold *a*, seed *s***Ensure:** 
Membership-weighted augmented set *W*  1:Estimate per-dimension variances and form the diagonal training-fold covariance matrix *D*   ▹ Equation (9)  2:Compute the central sample $\lambda_{cen}$ and partition counts $N_{F}$, $N_{C}$  3:Initialise $W\leftarrow {\left\{\left(\lambda_{k},1\right)\right\}}_{k=1}^{n}$  4:Initialise the pseudorandom generator with seed *s*  5:**while** $|W|<n+N_{\mathrm{aug}}$ **do**  6:      Draw a reference $\lambda_{k}$ and sample a target membership $\mu_{W}\geq a$  7:      Solve for the candidate $\lambda_{k}^{\prime }$ by attribution inversion                   ▹ Equation (12)  8:      Apply biased far/near acceptance with coefficients α,β                 ▹ Equation (13)  9:      **if** $\mu_{W}\left(\lambda_{k}^{\prime }\right)\geq a$ **then**10:           Assign a soft label by membership-weighted averaging over neighbours11:           $W\leftarrow W\cup \{\left(\lambda_{k}^{\prime },\mu_{W}\left(\lambda_{k}^{\prime }\right)\right)\}$12:      **end if**13:**end while**14:**return** *W*                                       ▹ Equation (14)

### 3.4. Classifier Training and Evaluation Protocol

Eight classifiers were evaluated on the fused GCNN–condition representation: Multi-Layer Perceptron (MLP), Decision Tree (DT), Random Forest (RF), Gradient Boosting Ensemble (GBE), XGBoost, Support Vector Classifier (SVC), K-Nearest Neighbours (KNN), and Logistic Regression. The unit of splitting was always an original experimental record. Virtual samples were training-fold derivatives and never served as validation units.

(i) Data splitting and cross-validation. Primary evaluation used stratified five-fold cross-validation defined on the 54 original experiments. Each outer split contained 43–44 training records and 10–11 untouched validation records. Within every split, descriptor scaling, supervised GCNN fitting, feature fusion, AAE generation, classifier selection, and probability calibration were fitted exclusively from the training fold. The validation fold was transformed once with the fitted scaler and encoder and was used only for metric calculation. For the AAE arm, virtual samples were generated after the split from the training records and their labels only. The reference arm followed the same fold assignments without virtual samples. Final metrics are reported as mean ± standard deviation over the five original-record validation folds. Six fixed seeds (7, 13, 21, 42, 73, and 97) were used for stochastic sensitivity analyses.
  Additional stress tests held out complete ligands, publication sources, or pH regions. Leave-one-ligand-out (LOLO) probes transfer to an unrepresented ligand identity; leave-one-publication-out (LOPO) limits source-specific duplication; and leave-region-out (LRO) probes condition-space shift. These grouped tests were analysed separately from random-fold cross-validation because their lower scores quantify a different and more difficult form of generalisation. A deliberately invalid augment-then-split protocol was retained only as a leakage control and was excluded from all model claims.
(ii) Evaluation metrics. Considering class imbalance, accuracy (ACC), F1 score, and the Matthews correlation coefficient (MCC) were adopted as primary metrics, complemented by the areas under the receiver-operating-characteristic and precision-recall curves (ROC-AUC and PR-AUC). The two threshold-independent metrics central to a balanced assessment are defined as
  (15) $$F_{1}=\frac{2\;TP}{2\;TP+FP+FN},$$
  (16) $$\mathrm{MCC}=\frac{TP\cdot TN-FP\cdot FN}{\sqrt{\left(TP+FP)(TP+FN)(TN+FP)(TN+FN\right)}},$$
  where TP,TN,FP,FN are the true/false positive/negative counts. MCC is reported because it remains informative under the strong class imbalance of the Au_25_ subset.
(iii) Interpretable model selection. Based on the comparison across classifiers, the DT was selected as the primary interpretable model owing to its strong performance on the augmented data and transparent decision structure. The tree was built with Gini impurity as the splitting criterion, and the maximum depth and minimum samples per leaf were constrained to mitigate overfitting. The resulting GCNN-AAE-DT model was used to extract explicit synthesis rules and condition windows for Au_25_ formation.

### 3.5. Implementation Details and Computational Cost

Molecular graphs and fingerprints were produced with RDKit and the DeepChem GraphConv featuriser. Within each outer training fold, each supervised component encoder used two graph-convolution layers with 64 hidden units per layer, followed by average pooling and a 128-unit dense layer. Batch normalisation was enabled and dropout was set to 0. The encoders were trained for 100 epochs with the Adam optimiser using a learning rate of 0.001 and a batch size of 32. The resulting embeddings were concatenated with the standardised process descriptors. The eight classifiers were implemented with scikit-learn and XGBoost under a single, shared feature pipeline, so that the only difference between the two schemes is the presence or absence of the AAE module. AAE itself is lightweight: because Equations (9)–(14) reduce to per-dimension variance estimation and closed-form attribution inversion, generating $2\times 10^{4}$ virtual samples from 54 records takes on the order of ten seconds on a commodity workstation, with candidate accounting and fixed seeds reported in Supplementary Table S2, and the cost grows roughly linearly with the requested sample count. This profile is attractive. Augmentation here is far cheaper than first-principles sampling, yet it stays physically constrained, which makes repeated and fold-consistent augmentation practical across the cross-validation and ablation experiments. To keep the comparison fair, hyperparameters were tuned only on training folds, and all randomness was controlled through fixed seeds so that the reported means and standard deviations are reproducible. A repository-level account of the augmentation procedure, including the descriptor post-processing and range-clipping rules, is provided as Supplementary Algorithm S1.

## 4. Conclusions

The strict fold-local analysis supports a narrower and more reproducible interpretation of AAE for small-sample gold-nanocluster synthesis modelling. The strongest no-augmentation MCC was 0.729, while the best AAE result was 0.758, corresponding to a modest local gain rather than near-perfect prediction. LOLO, LOPO, and LRO MCC values of 0.579, 0.526, and 0.652 further showed that transfer to unseen ligands, publication sources, and condition regions remained more difficult than random-fold interpolation. Feature ablation, calibration, covariance analysis, tree stability, SHAP, and symbolic regression converged on ligand aromaticity, temperature, and HAuCl_4_ concentration as the most reproducible factors. The resulting decision rules and condition maps can prioritise experiments within the represented chemical domain, but they do not replace targeted experimental validation or establish universal transfer beyond the 54-record dataset.

## Figures and Tables

**Table 1:** Performance comparison between Model without AAE and Model with AAE (mean ± standard deviation over 5-fold cross-validation).

Model	Classifier	ACC	F1	MCC
Without AAE	XGBoost	0.7782±0.0797	0.7741±0.0811	0.5957±0.1613
Without AAE	Logistic	0.8509±0.0536	0.8486±0.0561	0.7289±0.0974
Without AAE	SVC	0.7764±0.0581	0.7739±0.0574	0.5890±0.1399
Without AAE	RF	0.7400±0.0786	0.7310±0.0918	0.5257±0.1275
Without AAE	Decision Tree	0.8345±0.0366	0.8331±0.0368	0.6927±0.0748
Without AAE	KNN	0.7764±0.1079	0.7698±0.1135	0.5847±0.2142
Without AAE	GBE	0.7055±0.1146	0.6991±0.1212	0.4341±0.2229
Without AAE	MLP	0.5891±0.1535	0.5434±0.1819	0.1730±0.3973
Without AAE	Mean	0.7564	0.7466	0.5405
With AAE	XGBoost	0.8420±0.0660	0.8390±0.0680	0.6840±0.1390
With AAE	Logistic	0.8640±0.0470	0.8620±0.0490	0.7580±0.0910
With AAE	SVC	0.8180±0.0710	0.8150±0.0740	0.6510±0.1420
With AAE	RF	0.8360±0.0620	0.8330±0.0650	0.6710±0.1280
With AAE	Decision Tree	0.8460±0.0590	0.8430±0.0610	0.7240±0.1180
With AAE	KNN	0.8380±0.0740	0.8360±0.0760	0.7040±0.1490
With AAE	GBE	0.8490±0.0580	0.8470±0.0600	0.7180±0.1160
With AAE	MLP	0.8440±0.0670	0.8410±0.0710	0.7120±0.1370
With AAE	Mean	0.8421	0.8395	0.7027

**Table 2:** Relative performance improvement of Model with AAE over Model without AAE.

Classifier	ACC	F1	MCC
XGBoost	+8.2%	+8.4%	+14.8%
Logistic	+1.5%	+1.6%	+4.0%
SVC	+5.4%	+5.3%	+10.5%
RF	+13.0%	+14.0%	+27.6%
Decision Tree	+1.4%	+1.2%	+4.5%
KNN	+7.9%	+8.6%	+20.4%
GBE	+20.3%	+21.2%	+65.4%
MLP	+43.3%	+54.8%	+311.5%
Mean	+12.6%	+14.4%	+57.3%

**Table 3:** Mean F1 performance under different augmentation scales.

Scale	F1	Relative Improvement	Time (s)	Notes
AAE-2k	0.8390	+12.4%	3	Minimum scale provides an initial improvement under strict evaluation.
AAE-5k	0.8510	+14.0%	6	Performance continues to improve while variability decreases.
AAE-20k	0.8620	+15.4%	10	Best balance between performance and cost.
AAE-50k	0.8570	+14.8%	25	Performance declines slightly after the 20 k optimum.

## Data Availability

The code is available at https://github.com/youziyue0603/AuNCs-AAE (accessed on 28 July 2026). Data will be made available on request.

## Supplementary Materials

The following supporting information can be downloaded at https://www.mdpi.com/article/10.3390/molecules31162808/s1. Figures S1–S9: dataset overview, augmentation behaviour, strict classifier and scale comparisons, parameter sensitivity, leakage control, method ablations, grouped validation and calibration, and stability and explanation; Tables S1–S16: complete numerical results and audits; Algorithm S1: fold-local GCNN-AAE evaluation pipeline. External-record references used in Supplementary Table S12 are cited here [50,51,52,53,54,55,56,57].

## Abbreviations

The following abbreviations are used in this manuscript:

Au NC	Gold nanocluster
AAE	Approximate attribution enhancement
GCNN	Graph convolutional neural network
GNN	Graph neural network
SMILES	Simplified Molecular Input Line Entry System
DT	Decision tree
RF	Random forest
GBE	Gradient boosting ensemble
MLP	Multi-layer perceptron
SVC	Support vector classifier
KNN	K-nearest neighbors
RA	Reducing agent
ACC	Accuracy
MCC	Matthews correlation coefficient
ROC-AUC	Area under the receiver-operating-characteristic curve
PR-AUC	Area under the precision-recall curve
ECE	Expected calibration error
CASP	Computer-aided synthesis planning
CV	Cross-validation
DFT	Density functional theory

## Nomenclature

$G_{S}$	Molecular graph of a chemical species *S*
$h_{v},\;h_{i}^{\left(l\right)}$	Node (atom) feature vector; node feature at layer *l*
N(v)	Neighbour set of node *v*; deg(·) is node degree
$h^{\left(G\right)}$	Global GCNN embedding (molecular fingerprint)
$f_{s},\;f_{l},\;f_{r}$	Structural feature vectors of solvent, ligand, reducing agent
$\lambda^{\prime },\;\lambda_{k}$	Candidate condition–feature vector; *k*-th original vector
$\mu_{W}\left(\lambda^{\prime }|\lambda_{k}\right)$	Membership of candidate $\lambda^{\prime }$ relative to $\lambda_{k}$
n,d	Number of original samples in the current training fold; number of features
J	Index set of training-fold features with non-zero variance
*D*	Training-fold covariance matrix; diagonal in the principal AAE
$D^{-1}$	Precision matrix corresponding to *D*
$\sigma_{j}^{2}$	Population variance of feature *j* within the current training fold
*a*	Minimum membership threshold (*affi* in the implementation)
$N_{\mathrm{aug}}$	Target number of retained training-fold virtual samples
*s*	Random seed controlling stochastic candidate generation and selection
α,β	Attribution deviation coefficients (far/near acceptance)
ACC, F1, MCC	Accuracy, F1 score, Matthews correlation coefficient
