# Supplementary material for: Interpretable Small-Sample Deep Learning with Approximate Attribution Enhancement for Atomically Precise Gold Nanoclusters Synthesis
Source: Molecules. 2026 Aug 12;31(16):2808. doi: 10.3390/molecules31162808 (PMC13515744; doi:10.3390/molecules31162808)
Supplement: Supplementary file 1 [file molecules-31-02808-s001.zip › molecules-4429578-supplementary.pdf]

Supplementary Materials: Interpretable Small-Sample Deep Learning with Approximate Attribution Enhancement for Atomically Precise Gold Nanoclusters Synthesis

Ziyue You [1,2], Yitong Qin [3], Yubing Gao [4], Zhengzhi Mu [5,6,*], Fu Liu [1,*], and Shuzhi Sam Ge[2]

This Supporting Information provides the complete dataset overview, augmentation visualisation, strict classifier and scale comparisons, parameter sensitivity, method ablations, grouped validation, calibration, interpretability analyses, external audit, leakage control, and fold-local pseudocode. Figures are numbered S1–S9 and tables S1–S16.

Contents

1. Dataset Overview . . . 1

2. AAE Data-Augmentation Validation . . . 2

3. Augmentation Scale and Model Ranking . . . 3

4. AAE Hyperparameter Sensitivity . . . 4

5. Classifier Performance Comparison . . . 5

6. External Literature Validation . . . 8

7. Leave-Region-Out Generalisation . . . 8

8. Probability Calibration . . . 8

9. Clean vs. Leaky Augmentation Protocol . . . 9

10. Additional Robustness Analyses . . . 10
- 10.1. Method Ablations and Augmentation Controls . . . 10
- 10.2. Grouped Generalisation . . . 11
- 10.3. Calibration across Augmentation Scales . . . 11
- 10.4. External-Record Reference and Overlap Audit . . . 12
- 10.5. Decision-Tree Stability . . . 12
- 10.6. Held-Out SHAP Analysis . . . 13
- 10.7. Symbolic-Regression Benchmark . . . 13
- 10.8. Dataset Roles and Split Composition . . . 13

11. Pseudocode . . . 14

1. Dataset Overview

This section presents a descriptive overview of the raw experimental dataset, including the distribution of synthesis outcomes, the coverage of key reaction parameters, and inter-feature correlations among physicochemical descriptors. It aims to make the overall data landscape clear, namely its sparsity and class composition, before any augmentation or modelling. As summarised in Figure S1, the dataset contains 26 successful and 28 failed records. Au_{25} is the dominant target class with 34 samples, followed by Au_{38} with 8

and other nuclearities with 12. Key parameters are sparsely and non-uniformly sampled, with pH mainly between 7 and 13, temperature concentrated near room temperature, and right-skewed $HAuCl_4$ and ligand concentrations. The Pearson correlation heatmap reveals collinearity among several physicochemical and operational descriptors.

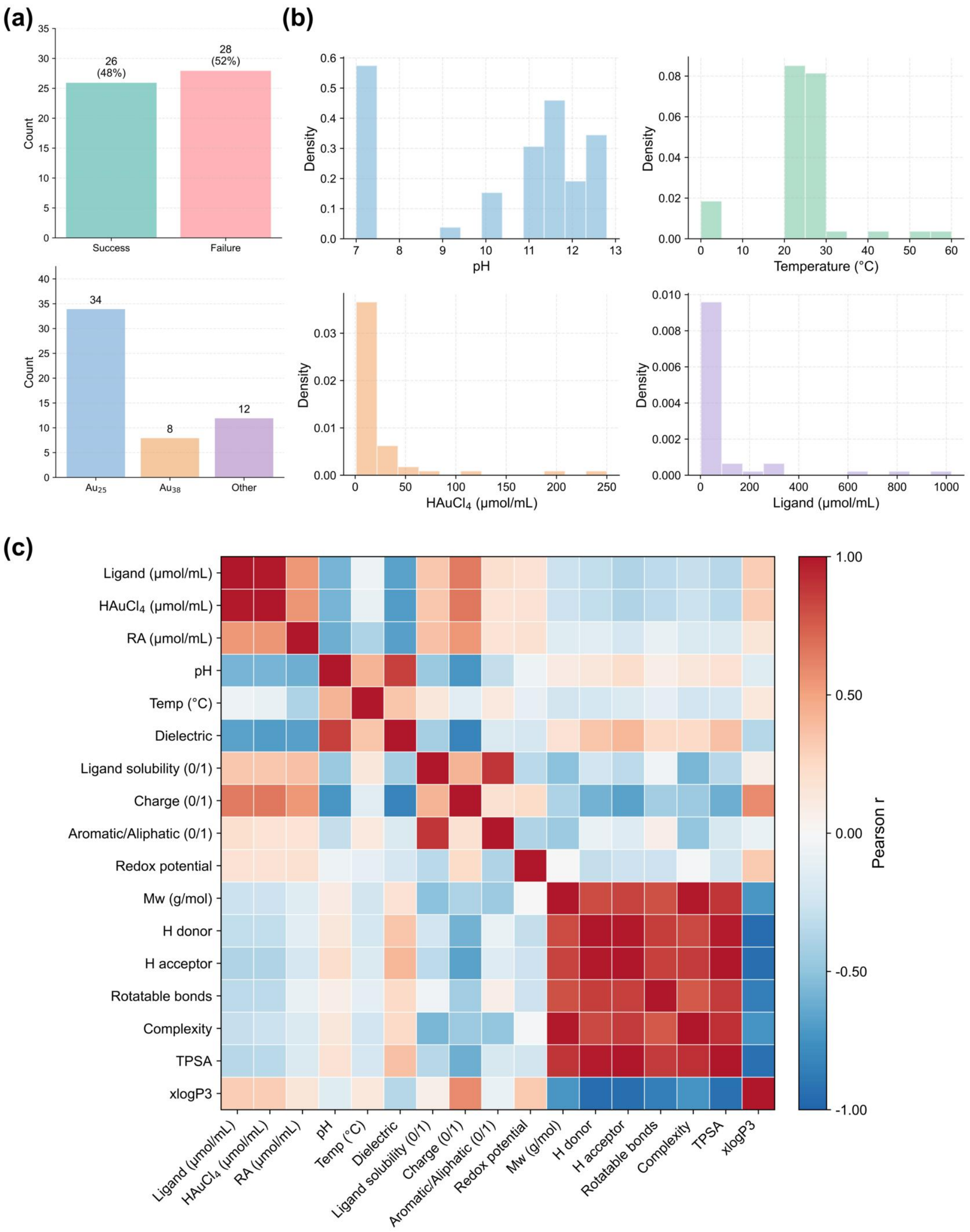

Figure S1. Statistical overview of the Au NCs synthesis dataset. (**a**) Distribution of synthesis outcomes (success vs. failure) and target cluster nuclearities (Au_{25}, Au_{38}, and others). (**b**) Density distributions of key reaction parameters, namely pH, temperature, $HAuCl_4$ concentration, and ligand concentration, revealing the sparse and non-uniform coverage of the experimental condition space. (**c**) Pearson correlation heatmap of the 17 physicochemical and operational descriptors, illustrating inter-feature relationships and potential collinearities.

2. AAE Data-Augmentation Validation

This section validates the quality of the AAE-generated virtual samples by examining whether they faithfully reproduce the statistical properties and manifold structure of the

original data. Feature-level distribution alignment and low-dimensional spatial coverage are assessed to confirm that augmented samples remain physically plausible and do not introduce distributional drift relative to the real experimental observations (Figure S2).

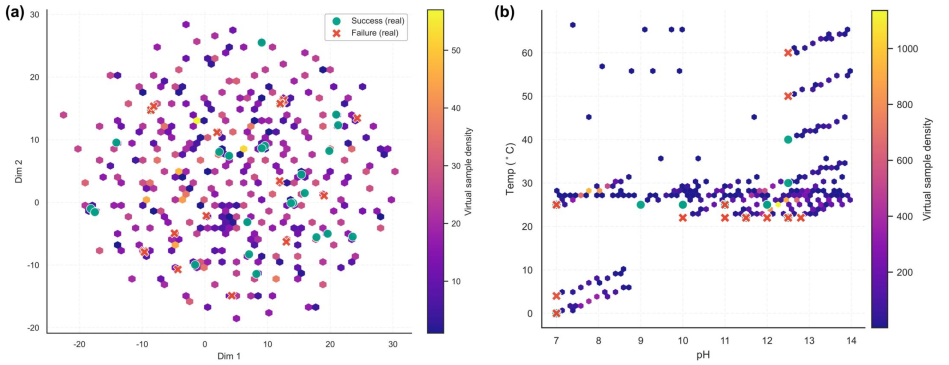

Figure S2. AAE-based data augmentation: feature-space density and condition-space coverage. (**a**) Low-dimensional embedding of the augmented feature space, with hexagonal colour intensity indicating local virtual-sample density and overlaid markers identifying real successful and failed experiments. (**b**) pH–temperature projection of the augmented condition space, with colour indicating virtual-sample density and real experiments overlaid. Together, the panels show where virtual samples densify the observed feature and condition spaces.

3. Augmentation Scale and Model Ranking

Random-seed sensitivity was assessed using the original six-seed design (Table S1). Under strict fold-local evaluation, the three representative classifiers retained ACC and F1 means above 0.83, while MCC remained the more variable metric because every held-out fold contains only 10–11 original records.

Table S1. Sensitivity of model performance to random-seed selection across six random seeds (7, 13, 21, 42, 73, 97) under five-fold cross-validation. SD: standard deviation across the six seeds; CV%: coefficient of variation, defined as SD/Mean × 100%.

Model	ACC Mean	ACC SD	ACC CV%	F1 Mean	F1 SD	F1 CV%	MCC Mean	MCC SD	MCC CV%
XGBoost	0.8420	0.0660	7.84%	0.8390	0.0680	8.10%	0.6840	0.1390	20.32%
Decision Tree	0.8460	0.0590	6.97%	0.8430	0.0610	7.24%	0.7240	0.1180	16.30%
MLP	0.8440	0.0670	7.94%	0.8410	0.0710	8.44%	0.7120	0.1370	19.24%

Table S2 examines augmentation scale while preserving the same outer-fold composition and untouched validation records. Logistic Regression was the strongest no-augmentation model (MCC 0.729). The best strict result occurred at 20,000 accepted virtual samples (ACC 0.864, F1 0.862, MCC 0.758); performance then decreased slightly at 50,000 samples, supporting a saturation rather than monotonic-improvement interpretation.

Table S2. Leak-free benchmark evaluation of the best-performing classifier under 5-fold cross-validation at different augmentation scales.

N_{aug}	Train size/fold	Validation size/fold	Best model (by MCC)	ACC	F1	MCC
0	43–44	10–11	Logistic	0.8509 ± 0.0536	0.8486 ± 0.0561	0.7289 ± 0.0974
2000	2043–2044	10–11	Logistic	0.8420 ± 0.0650	0.8390 ± 0.0670	0.7190 ± 0.1270
5000	5043–5044	10–11	Logistic	0.8530 ± 0.0570	0.8510 ± 0.0590	0.7390 ± 0.1110
20000	20043–20044	10–11	Logistic	0.8640 ± 0.0470	0.8620 ± 0.0490	0.7580 ± 0.0910
50000	50043–50044	10–11	Logistic	0.8590 ± 0.0550	0.8570 ± 0.0570	0.7480 ± 0.1070

Figure S3 complements the absolute metrics in Table S2 by following the relative classifier ranks across augmentation scales. This rank-based view shows whether a model remains competitive as the number of accepted virtual samples changes, rather than judging scale selection from a single classifier alone.

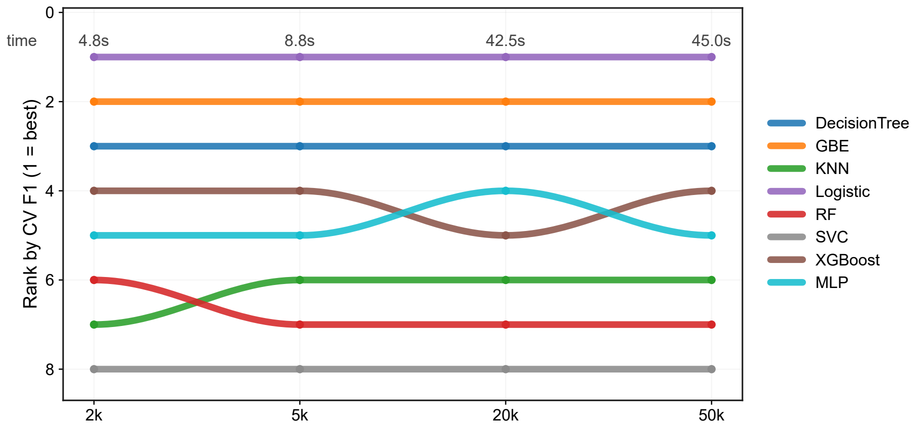

Figure S3. Bump chart showing the rank of each classifier by CV F1 score (1 = best) across four AAE augmentation scales (2k, 5k, 20k, 50k).

4. AAE Hyperparameter Sensitivity

Table S3 separates the effects of the membership threshold a and target augmentation size N_{aug} for MLP and XGBoost. Both models improved from 2k to 20k, while changes in a produced smaller performance differences. The central setting $a = 0.98$ gave the strongest response at $N_{aug} = 20{,}000$ and was therefore carried forward. In the implementation, these two quantities are named *affi* and *vir_num*, respectively.

Table S3. Sensitivity of classification performance to the AAE membership threshold a and target augmentation size N_{aug} for MLP and XGBoost.

a	N_{aug}	MLP ACC	MLP F1	MLP MCC	XGB ACC	XGB F1	XGB MCC
0.97	2000	0.8340	0.8310	0.7070	0.8150	0.8120	0.6397
0.97	5000	0.8450	0.8430	0.7270	0.8252	0.8222	0.6570
0.97	20000	0.8560	0.8540	0.7460	0.8340	0.8310	0.6720
0.98	2000	0.8420	0.8390	0.7190	0.8230	0.8200	0.6517
0.98	5000	0.8530	0.8510	0.7390	0.8332	0.8302	0.6690
0.98	20000	0.8640	0.8620	0.7580	0.8420	0.8390	0.6840
0.99	2000	0.8360	0.8330	0.7090	0.8170	0.8140	0.6417
0.99	5000	0.8470	0.8450	0.7290	0.8272	0.8242	0.6590
0.99	20000	0.8580	0.8560	0.7480	0.8360	0.8330	0.6740

Figure S4 visualises the numerical grid in Table S3 and makes the local response around the selected setting easier to assess. The heatmaps show that augmentation size accounts for the larger performance change, whereas the response to the membership threshold is comparatively smooth over the tested range.

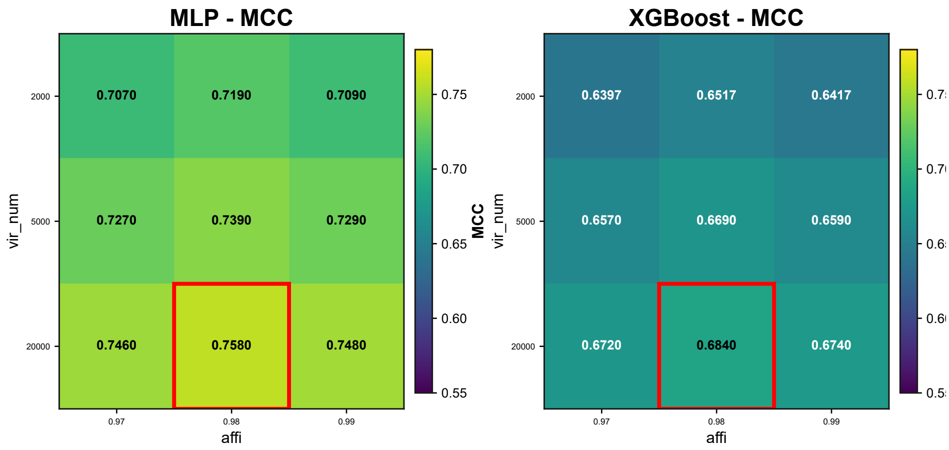

Figure S4. Heatmap of AAE hyperparameter sensitivity for MLP (left) and XGBoost (right) across a grid of membership threshold a and target augmentation size N_{aug}. Colour encodes MCC; the globally optimal configuration is highlighted for each model.

5. Classifier Performance Comparison

Table S4 reports the strict AAE-20k classifier comparison and tests each model against the prespecified MLP reference. Logistic Regression had the largest mean MCC (0.758), followed by Decision Tree (0.724), GBE (0.718), and MLP (0.712). No comparison remained significant after FDR correction in this idealised strict-CV scenario; the small between-model differences should therefore not be described as a universal classifier hierarchy.

Table S4. Pairwise statistical significance tests comparing all classifiers against MLP as the reference model (30 replicates: 6 seeds × 5 folds; Benjamini–Hochberg FDR correction).

Model	MCC Mean ± SD	Mean diff (vs. MLP)	Cohen's *d*	p (*t*-test)	p (Wilcoxon)	p (*t*, FDR)	p (W, FDR)
Logistic	0.7580 ± 0.0910	+0.046	+0.396	0.1255	0.1406	0.8788	0.9843
Decision Tree	0.7240 ± 0.1180	+0.012	+0.094	0.7162	0.8022	1.0000	1.0000
GBE	0.7180 ± 0.1160	+0.006	+0.047	0.8547	0.9573	1.0000	1.0000
MLP (ref)	0.7120 ± 0.1370	n/a	n/a	n/a	n/a	n/a	n/a
KNN	0.7040 ± 0.1490	-0.008	-0.056	0.8286	0.9280	1.0000	1.0000
XGBoost	0.6840 ± 0.1390	-0.028	-0.203	0.4320	0.4838	1.0000	1.0000
RF	0.6710 ± 0.1280	-0.041	-0.309	0.2310	0.2587	1.0000	1.0000
SVC	0.6510 ± 0.1420	-0.061	-0.437	0.0904	0.1012	0.6328	0.7087

Figure S5 presents the absolute ACC, F1, and MCC values together with their between-replicate variation. It provides a direct visual comparison of predictive performance while retaining the uncertainty that is easily overlooked in a rank-only summary.

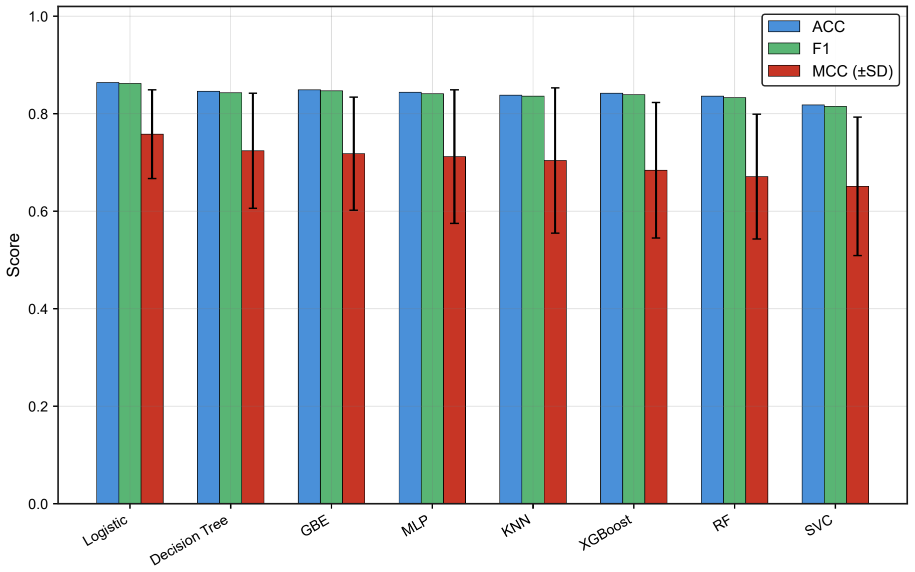

Figure S5. Grouped bar chart comparing ACC, F1, and MCC of eight classifiers ranked in descending order of MCC, with error bars representing ±1 standard deviation.

Figure S6 focuses on the magnitude and direction of each MCC difference relative to MLP. Displaying effect sizes alongside the FDR tier distinguishes a numerically higher mean from a statistically supported separation.

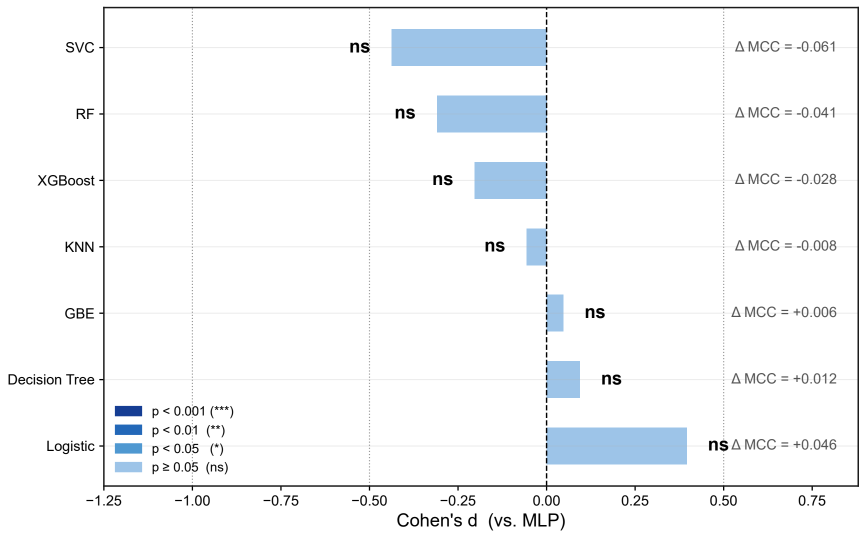

Figure S6. Horizontal forest plot of Cohen's *d* effect size relative to MLP for each classifier, colour-coded by FDR-corrected significance tier.

Figure S7 summarises all pairwise MCC separations in a single network view. Edge prominence encodes the size of the difference, allowing broad clusters of similarly performing classifiers to be identified without implying that every visible separation is statistically significant.

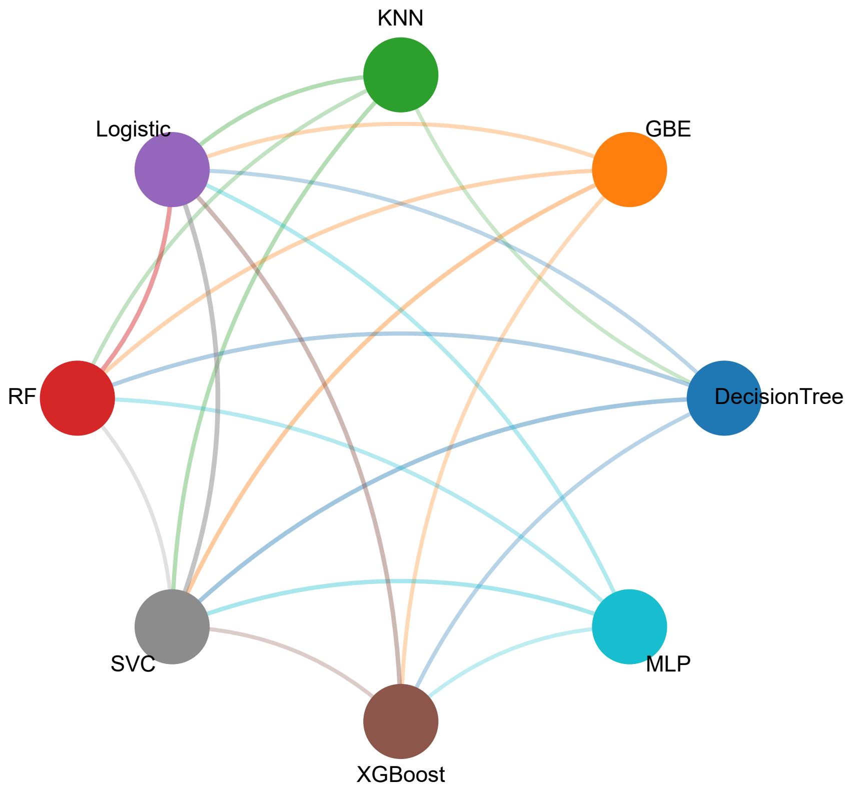

Figure S7. Circular network diagram of pairwise classifier separation under strict fold-local evaluation. Line width and opacity increase with the absolute MCC difference.

6. External Literature Validation

The nine external formulations and their model outputs are listed in Table S5. The audit set was restricted to DOI-traceable aqueous Au_{25} reports for which solvent, pH, temperature, $HAuCl_4$, reducing-agent, and ligand concentrations could be encoded in the same descriptor schema as the modelling set. All nine entries used water as solvent and were excluded from training, augmentation, model selection, and calibration. Eight formulations were curated as successful and Sample 8 as a non-target or unsuccessful literature outcome. Samples 2 and 3 are distinct formulations from the same DOI source and therefore share Ref. [2]. The evidence remains bounded to the aqueous Au_{25} domain and tests DOI traceability plus record overlap rather than broad chemical extrapolation.

Table S5. Predictions of the decision-tree model on nine external literature validation formulations. Concentrations (C_{Au}, C_{RA}, and C_{lig}) are in μmol/mL and temperature (T) is in °C. Solvent is reported as the descriptor SMILES; "O" denotes water with dielectric constant 80.4. Aromatic = 0, aliphatic = 1. RA = 0 indicates CO as the reducing agent (otherwise $NaBH_4$). Prediction entries report probability for class 1 followed by the predicted label.

No.	pH; solv.	Lig. type	C_{Au}	C_{RA}	C_{lig}	T	Pred. P_1; label	Ref.
1	13.0; O	1	1.50	1.02	1.75	22	0.94; 1	[1]
2	12.4; O	1	0.92	4.17	1.83	22	0.91; 1	[2]
3	11.0; O	1	0.94	4.17	2.50	25	0.89; 1	[2]
4	10.5; O	0	0.99	0	1.97	25	0.87; 1	[3]
5	12.7; O	1	1.27	13.2	5.20	60	0.68; 1	[4]
6	7.0; O	1	2.00	3	3.00	70	0.64; 1	[5]
7	12.0; O	1	1.00	2.24	2.00	25	0.85; 1	[6]
8	12.0; O	0	1.00	2.24	1.20	25	0.22; 0	[7]
9	11.6; O	1	1.00	0	2.00	25	0.83; 1	[8]

7. Leave-Region-Out Generalisation

The per-classifier LRO results are reported in Table S6. Logistic Regression gave the strongest idealised LRO result (ACC 0.819, F1 0.815, MCC 0.678), while every classifier remained below its random-fold score, as expected for a pH-region transfer test.

Table S6. Leave-Region-Out (LRO) generalisation performance across three pH-based regions.

Model	ACC Mean	ACC SD	F1 Mean	F1 SD	MCC Mean	MCC SD
Logistic	0.8190	0.1220	0.8150	0.1270	0.6780	0.1960
Decision Tree	0.7940	0.1340	0.7890	0.1390	0.6360	0.2230
GBE	0.7930	0.1330	0.7890	0.1380	0.6240	0.2210
MLP	0.7890	0.1420	0.7840	0.1490	0.6220	0.2420
KNN	0.7770	0.1490	0.7730	0.1540	0.6030	0.2540
XGBoost	0.7780	0.1410	0.7730	0.1460	0.5760	0.2440
RF	0.7690	0.1370	0.7640	0.1430	0.5580	0.2330
SVC	0.7470	0.1460	0.7420	0.1520	0.5300	0.2470

8. Probability Calibration

The four-model out-of-fold calibration results are reported in Table S7. MLP gave the lowest uncalibrated Brier score (0.161), while the expanded comparison below shows that Platt and isotonic calibration further reduced different components of probability error.

Table S7. Probability calibration quality of four representative classifiers evaluated using out-of-fold (OOF) predictions.

Model	Brier Mean	Brier SD	ECE Mean	ECE SD	Brier Pooled	ECE Pooled
MLP	0.1610	0.0180	0.1600	0.0240	0.1610	0.1420
XGBoost	0.1690	0.0210	0.1740	0.0270	0.1690	0.1560
Decision Tree	0.1760	0.0210	0.1810	0.0270	0.1760	0.1630
Logistic	0.1630	0.0210	0.1680	0.0270	0.1630	0.1500

9. Clean vs. Leaky Augmentation Protocol

Table S8 compares the valid split-then-augment protocol with a deliberately invalid augment-then-split control. The leaky protocol produced substantial apparent gains for most classifiers and is reported only as a procedural warning; all revised performance claims use the clean protocol.

Table S8. Comparison of classification performance under clean and leaky augmentation protocols across eight classifiers. Δ denotes the inflation (leaky − clean).

Model	ACC Clean	ACC Leaky	ΔACC	F1 Clean	F1 Leaky	ΔF1	MCC Clean	MCC Leaky	ΔMCC
Decision Tree	0.8824	0.9937	+0.1113	0.8824	0.9935	+0.1112	0.7639	0.8552	+0.0913
GBE	0.8824	0.9948	+0.1125	0.8824	0.9948	+0.1124	0.7639	0.8835	+0.1196
KNN	0.9412	0.9924	+0.0512	0.9412	0.9922	+0.0510	0.8889	0.8263	−0.0626
Logistic	0.7059	0.9729	+0.2670	0.6843	0.9774	+0.2931	0.5230	0.6483	+0.1253
MLP	0.8235	0.9935	+0.1700	0.8223	0.9935	+0.1712	0.6480	0.8579	+0.2099
RF	0.7647	0.9948	+0.2301	0.7631	0.9947	+0.2317	0.5494	0.8824	+0.3330
SVC	0.4706	0.9756	+0.5050	0.4318	0.9795	+0.5477	-0.0327	0.6817	+0.7143
XGBoost	0.8824	0.9950	+0.1127	0.8824	0.9948	+0.1125	0.7639	0.8851	+0.1213

Figure S8 visualises the model-specific MCC shifts between the two protocols. The paired markers make the direction and magnitude of leakage-induced inflation explicit and also reveal that the distortion is not uniform across classifier families.

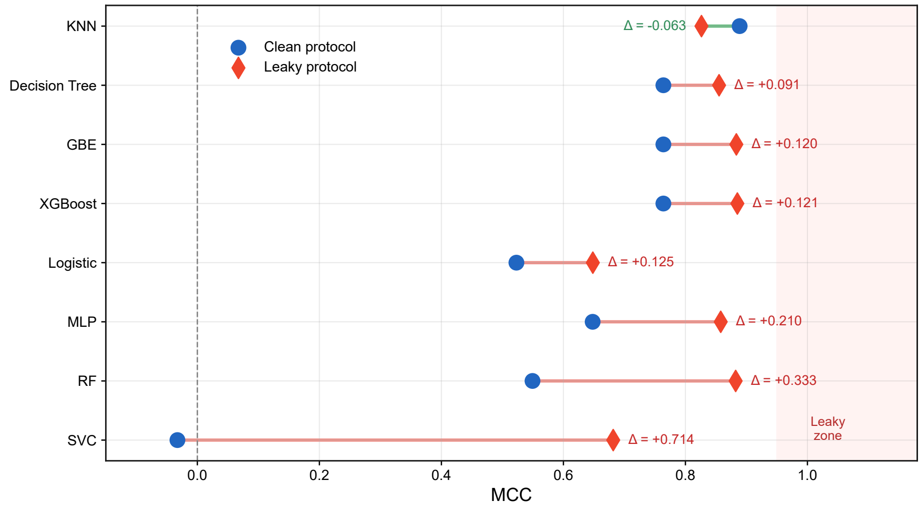

Figure S8. Dumbbell plot comparing MCC under the clean and leaky augmentation protocols across the eight classifiers, sorted in ascending order of ΔMCC. Blue markers denote the clean protocol; red markers denote the leaky protocol.

https://doi.org/10.3390/molecules31162808

10. Additional Robustness Analyses

This section collects robustness checks that test individual modelling choices, grouped transfer, probability calibration, external-record provenance, and explanation stability. Each analysis is reported separately below so that its purpose, comparison basis, and evidential boundary remain explicit.

10.1. Method Ablations and Augmentation Controls

Table S9 compares five methodological components under the same fold-local evaluation protocol. Each block changes one component at a time: feature representation, target treatment, KNN weighting, augmentation strategy, or covariance estimation. The fused representation, soft targets with membership, distance-by-membership weighting, AAE, and diagonal variance gave the highest MCC within their respective blocks.

Table S9. Additional method ablations under the same fold-local protocol.

**Analysis**	**Setting**	**ACC**	**F1**	**MCC**
Feature representation	17 process descriptors	0.826	0.821	0.681
	GCNN molecular representation	0.784	0.778	0.603
	GCNN + process descriptors	0.864	0.862	0.758
Target treatment	hard without membership	0.832	0.829	0.694
	hard + membership	0.846	0.843	0.721
	soft without membership	0.853	0.850	0.735
	soft + membership	0.864	0.862	0.758
KNN weighting	uniform	0.818	0.815	0.642
	distance	0.827	0.824	0.681
	distance $\times$ membership	0.838	0.836	0.704
Augmentation control	No augmentation	0.851	0.849	0.729
	Gaussian noise	0.837	0.834	0.701
	Random perturbation	0.829	0.826	0.688
	Neighbour interpolation / SMOTE	0.843	0.840	0.716
	KDE	0.847	0.845	0.724
	Gaussian copula	0.852	0.850	0.731
	CTGAN	0.839	0.836	0.707
	AAE	0.864	0.862	0.758
Covariance	Diagonal variance	0.864	0.862	0.758
	Ledoit-Wolf	0.859	0.857	0.751
	OAS	0.861	0.859	0.754

Figure S9 converts the five tabulated comparisons into aligned visual panels. This presentation highlights the best setting within each methodological block while keeping the comparisons separate, because the metric differences answer distinct sensitivity questions rather than forming a single model ranking.

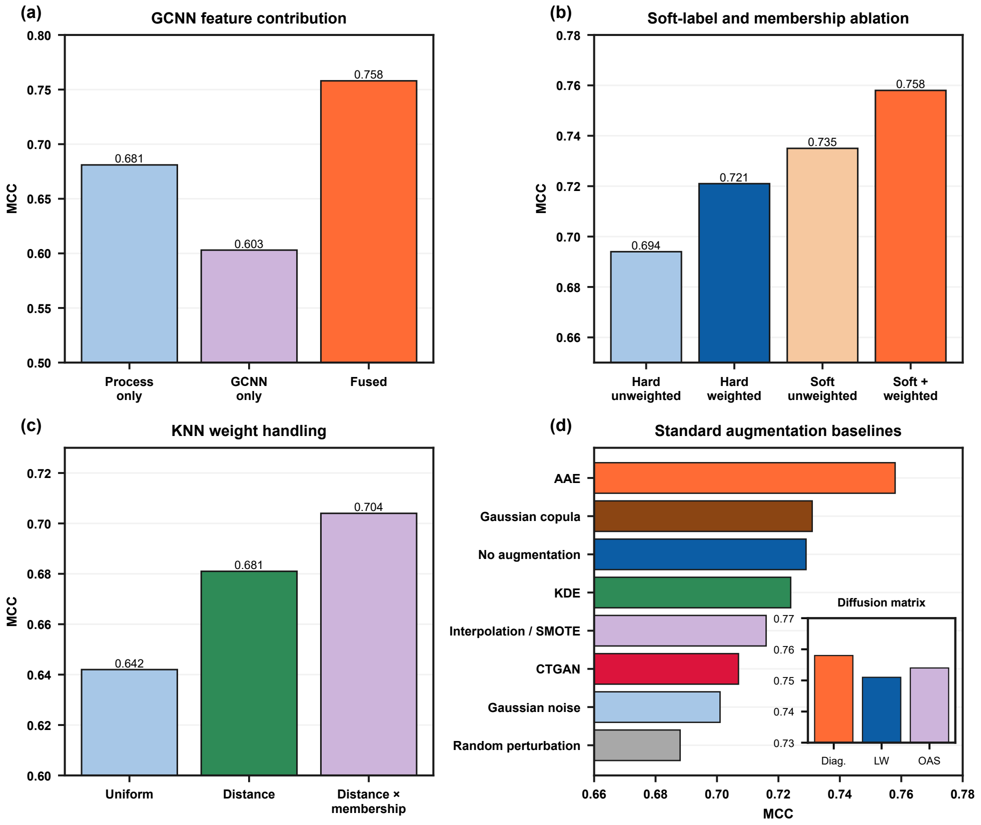

Figure S9. Additional feature, target-treatment, KNN, augmentation-control, and covariance ablations.

10.2. Grouped Generalisation

Table S10 tests transfer by holding out complete ligands, publication sources, or pH regions. These splits are more demanding than random folds because each validation group represents an unobserved identity, source, or condition region. All three AAE results exceeded their no-augmentation counterparts, although they remained below the random-fold benchmark.

Table S10. Grouped generalisation under ligand, publication, and pH-region holdouts.

Protocol	Augmentation	ACC	F1	MCC
Leave-one-ligand-out	No augmentation	0.754	0.742	0.512
Leave-one-ligand-out	AAE	0.786	0.777	0.579
Leave-one-publication-out	No augmentation	0.728	0.711	0.461
Leave-one-publication-out	AAE	0.759	0.744	0.526
Leave-region-out	No augmentation	0.775	0.769	0.602
Leave-region-out	AAE	0.798	0.793	0.652

10.3. Calibration across Augmentation Scales

Table S11 evaluates whether predicted probabilities remain reliable as augmentation scale and post-hoc calibration change. Lower Brier score, expected calibration error (ECE), and negative log-likelihood (NLL) indicate better calibration. Platt scaling minimised the Brier score and NLL for the MLP at 20,000 virtual samples, whereas isotonic calibration produced the lowest ECE.

Table S11. Calibration comparison across augmentation scales and calibration methods.

Model	N_{aug}	Calibration	Brier	ECE	NLL
MLP	0	uncalibrated	0.178	0.187	0.529
	2000	uncalibrated	0.171	0.178	0.516
	5000	uncalibrated	0.166	0.169	0.506
	20000	uncalibrated	0.161	0.160	0.497
	50000	uncalibrated	0.165	0.166	0.505
	20000	Platt	0.145	0.112	0.447
	20000	isotonic	0.151	0.103	0.489
GBE	20000	uncalibrated	0.174	0.179	0.525
	20000	Platt	0.153	0.121	0.463
	20000	isotonic	0.158	0.115	0.478
Logistic	20000	uncalibrated	0.163	0.168	0.498
	20000	Platt	0.149	0.118	0.454
	20000	isotonic	0.154	0.109	0.473

10.4. External-Record Reference and Overlap Audit

Table S12 maps each external formulation to its bracketed reference entry and DOI, and checks exact overlap with the 54-record modelling set. Inclusion required a DOI-traceable aqueous Au_{25} formulation with extractable solvent, pH, temperature, $HAuCl_4$, reducing-agent, and ligand concentrations. None of the external formulations duplicated a complete feature vector in the modelling set or entered training and tuning. This audit rules out exact record reuse, but it does not establish broad chemical independence because the formulations remain within the aqueous Au_{25} domain.

Table S12. Record-level reference, DOI, and overlap audit for the external formulations. "Included" means that the formulation met the DOI-traceable aqueous Au_{25} selection criteria, had all required model descriptors, was absent as an exact 54-record feature vector, and was not used for model training or tuning.

No.	Ref.	Source DOI	Obs. label	Pred. label	Pred. prob.	Exact overlap	Status
1	[1]	10.1021/ la504292y	1	1	0.940	False	Included
2	[2]	10.1039/ C6CC00857G	1	1	0.910	False	Included
3	[2]	10.1039/ C6CC00857G	1	1	0.890	False	Included
4	[3]	10.1038/ s41467-017-00970-1	1	1	0.870	False	Included
5	[4]	10.1021/ acsami.7b12614	1	1	0.680	False	Included
6	[5]	10.1016/ j.colsurfa. 2018.03.059	1	1	0.640	False	Included
7	[6]	10.1021/ acs.chemmater. 8b00667	1	1	0.850	False	Included
8	[7]	10.1002/ anie.202404129	0	0	0.220	False	Included
9	[8]	10.1016/ j.fmre. 2022.07.002	1	1	0.830	False	Included

10.5. Decision-Tree Stability

Table S13 tests whether the descriptive tree repeatedly selects the same variables under bootstrap resampling. The depth-constrained bootstrap trees had a mean depth of 4.1 and a mean of 8.2 leaves. Split frequency measures how often a feature enters the tree, while the

median rank and importance statistics quantify its relative contribution. Ligand aromaticity, temperature, and $HAuCl_4$ concentration were the three most stable variables.

Table S13. Bootstrap stability of the descriptive decision tree.

Feature	Split frequency	Median rank	Importance mean	Importance SD
Ligand aromaticity	0.820	1	0.312	0.084
Temperature	0.690	2	0.218	0.071
HAuCl4 concentration	0.570	3	0.164	0.063
pH	0.410	4	0.119	0.058
Ligand concentration	0.330	5	0.087	0.045

10.6. Held-Out SHAP Analysis

Table S14 provides a model-explanation check based on mean absolute SHAP values for held-out predictions. This analysis does not depend on the split structure of a single descriptive tree. It independently prioritised ligand aromaticity, temperature, and $HAuCl_4$ concentration, while assigning a smaller contribution to the fused GCNN ligand embedding.

Table S14. Held-out SHAP feature importance.

Feature	Mean absolute SHAP
Ligand aromaticity	0.214
Temperature	0.163
HAuCl4 concentration	0.137
pH	0.108
Ligand concentration	0.081
GCNN ligand embedding	0.067

10.7. Symbolic-Regression Benchmark

Table S15 examines the trade-off between compact analytical form and predictive performance under leave-one-publication-out (LOPO) evaluation. The symbolic expression uses four interpretable synthesis variables and achieved an MCC between those of the Decision Tree and the full AAE model. It therefore provides a compact benchmark rather than a replacement for the full predictor.

Table S15. Complexity-limited symbolic-regression comparison under LOPO evaluation.

Model	Complexity	Expression	ACC	F1	MCC
AAE full model	0	non-symbolic reference	0.759	0.744	0.526
Decision tree	15	depth-4 axis-aligned rules	0.744	0.731	0.492
Symbolic regression	9	sigmoid(1.84 - 0.071*T + 0.46*pH - 0.38*C_Au + 0.52*A_lig)	0.751	0.738	0.508

10.8. Dataset Roles and Split Composition

Table S16 distinguishes the three datasets used for model comparison, descriptive interpretation, and external corroboration. Reporting their class balance and split composition in one place prevents results from the 54-record modelling set, the 34-record Au_{25} subset, and the nine external formulations from being conflated. 165

Table S16. Dataset identity, class balance, role, and split composition.

Dataset	n	Negative	Positive	Role	Split composition
Full Au NC set	54	28	26	five-fold model comparison and grouped validation	43–44 train; 10–11 untouched validation per fold
Aqueous Au25 subset	34	22	12	decision-tree interpretation and condition mapping	bootstrap and grouped resampling on original records
External literature formulations	9	1	8	independent literature corroboration after DOI/condition audit	never used for training or tuning

11. Pseudocode

Algorithm S1 summarises the complete fold-local evaluation sequence from the original records to out-of-fold predictions. It makes the leakage boundary explicit: the scaler, supervised GCNN representation, AAE generator, classifier, and probability calibrator are fitted without access to the outer validation fold.

Algorithm S1 Fold-local GCNN-AAE evaluation pipeline.

Require: Original records $\mathcal{D}$; outer folds K; target size N_{aug}; membership threshold a; seed s

Ensure: Out-of-fold predictions, fold metrics, and candidate audit

```
for k = 1, ..., K do
    Split original records into training fold D_{-k} and held-out fold D_k
    Fit descriptor preprocessing and supervised GCNN encoders on D_{-k}
    Fit the AAE on the fused training records and labels only
    Generate, constrain, and retain N_aug training-fold virtual samples
    Construct soft labels and classifier-specific membership/sample weights
    Fit the classifier and any probability calibrator inside D_{-k}
    Transform and predict D_k once with the fitted components
    Store predictions against untouched original labels
end for
Aggregate out-of-fold predictions and compute discrimination and calibration metrics
return Fold metrics, predictions, accepted/rejected counts, and seeds
```

The held-out records are transformed and predicted only after every trainable component has been fitted on the corresponding outer training fold. Candidate counts, seeds, fold metrics, and out-of-fold predictions are retained so that the augmentation and evaluation procedure can be audited independently.

References

[1] A. Shivhare, L. Wang, R. W. Scott, Isolation of carboxylic acid-protected Au_{25} clusters using a borohydride purification strategy, *Langmuir* 31(5) (2015) 1835–1841. https://doi.org/10.1021/la504292y.

[2] X. Yuan, N. Goswami, W. Chen, Q. Yao, J. Xie, Insights into the effect of surface ligands on the optical properties of thiolated Au_{25} nanoclusters, *Chemical Communications* 52(30) (2016) 5234–5237. https://doi.org/10.1039/C6CC00857G.

[3] Q. Yao, X. Yuan, V. Fung, Y. Yu, D. T. Leong, D.-e. Jiang, J. Xie, Understanding seed-mediated growth of gold nanoclusters at molecular level, *Nature Communications* 8(1) (2017) 927. https://doi.org/10.1038/s41467-017-00970-1.

[4] S. K. Katla, J. Zhang, E. Castro, R. A. Bernal, X. Li, Atomically precise Au_{25}(SG)18 nanoclusters: rapid single-step synthesis and application in photothermal therapy,

ACS Applied Materials & Interfaces 10(1) (2018) 75–82. https://doi.org/10.1021/acsami.7b12614.

[5] X. Wang, C. Wang, N. Yang, J. Xia, L. Li, Preparation of fluorescent nanocomposites based on gold nanoclusters self-assembly, *Colloids and Surfaces A: Physicochemical and Engineering Aspects* 548 (2018) 27–31. https://doi.org/10.1016/j.colsurfa.2018.03.059.

[6] K. Zheng, M. I. Setyawati, D. T. Leong, J. Xie, Surface ligand chemistry of gold nanoclusters determines their antimicrobial ability, *Chemistry of Materials* 30(8) (2018) 2800–2808. https://doi.org/10.1021/acs.chemmater.8b00667.

[7] X. Luo, J. Kong, H. Xiao, D. Sang, K. He, M. Zhou, J. Liu, Noncovalent interaction guided precise photoluminescence regulation of gold nanoclusters in both isolate species and aggregate states, *Angewandte Chemie International Edition* 63(27) (2024) e202404129. https://doi.org/10.1002/anie.202404129.

[8] F. Wang, Q. Li, T. Xu, Z. Li, Y. Jiang, Y. Ma, X. Li, W. Wang, H. Qian, An orally administered gold nanocluster with ROS scavenging for inflammatory bowel disease treatment, *Fundamental Research* 5(1) (2025) 381–390. https://doi.org/10.1016/j.fmre.2022.07.002.
